# Significance of molecular classification of ependymomas: *C11orf95-RELA* fusion-negative supratentorial ependymomas are a heterogeneous group of tumors

**DOI:** 10.1186/s40478-018-0630-1

**Published:** 2018-12-04

**Authors:** Kohei Fukuoka, Yonehiro Kanemura, Tomoko Shofuda, Shintaro Fukushima, Satoshi Yamashita, Daichi Narushima, Mamoru Kato, Mai Honda-Kitahara, Hitoshi Ichikawa, Takashi Kohno, Atsushi Sasaki, Junko Hirato, Takanori Hirose, Takashi Komori, Kaishi Satomi, Akihiko Yoshida, Kai Yamasaki, Yoshiko Nakano, Ai Takada, Taishi Nakamura, Hirokazu Takami, Yuko Matsushita, Tomonari Suzuki, Hideo Nakamura, Keishi Makino, Yukihiko Sonoda, Ryuta Saito, Teiji Tominaga, Yasuhiro Matsusaka, Keiichi Kobayashi, Motoo Nagane, Takuya Furuta, Mitsutoshi Nakada, Yoshitaka Narita, Yuichi Hirose, Shigeo Ohba, Akira Wada, Katsuyoshi Shimizu, Kazuhiko Kurozumi, Isao Date, Junya Fukai, Yousuke Miyairi, Naoki Kagawa, Atsufumi Kawamura, Makiko Yoshida, Namiko Nishida, Takafumi Wataya, Masayoshi Yamaoka, Naohiro Tsuyuguchi, Takehiro Uda, Mayu Takahashi, Yoshiteru Nakano, Takuya Akai, Shuichi Izumoto, Masahiro Nonaka, Kazuhisa Yoshifuji, Yoshinori Kodama, Masayuki Mano, Tatsuya Ozawa, Vijay Ramaswamy, Michael D. Taylor, Toshikazu Ushijima, Soichiro Shibui, Mami Yamasaki, Hajime Arai, Hiroaki Sakamoto, Ryo Nishikawa, Koichi Ichimura, on behalf of the Japan Pediatric Molecular Neuro-Oncology Group (JPMNG)

**Affiliations:** 1 Division of Brain Tumor Translational Research, National Cancer Center Research Institute, 5-1-1 Tsukiji, Chuo-ku, Tokyo, 104-0045 Japan; 2 Department of Neuro-Oncology/Neurosurgery, Saitama Medical University International Medical Center, Hidaka, Saitama Japan; 3 Department of Biomedical Research and Innovation, Institute for Clinical Research, Osaka National Hospital, National Hospital Organization|, Osaka, Japan; 4 Department of Neurosurgery, Osaka National Hospital, National Hospital Organization, Osaka, Japan; 5 Division of Epigenomics, National Cancer Center Research Institute, Tokyo, Japan; 6 Department of Bioinformatics, National Cancer Center Research Institute, Tokyo, Japan; 7 Department of Clinical Genomics, National Cancer Center Research Institute, Tokyo, Japan; 8 Division of Genome Biology, National Cancer Center Research Institute, Tokyo, Japan; 9 Department of Pathology, Saitama Medical University, Saitama, Japan; 10 Department of Pathology, Gunma University Hospital, Maebashi, Gunma Japan; 11 Department of Diagnostic Pathology, Hyogo Cancer Center, Kobe, Hyogo Japan; 12 Department of Laboratory Medicine and Pathology (Neuropathology), Tokyo Metropolitan Neurological Hospital, Tokyo, Japan; 13 Department of Pathology and Clinical Laboratories, National Cancer Center Hospital, Tokyo, Japan; 14 Department of Pediatric Hematology and Oncology, Osaka City General Hospital, Osaka, Japan; 15 Department of Neurosurgery, Graduate School of Medicine, Yokohama City University, Fukuura, Kanagawa Japan; 16 Department of Neurosurgery, Faculty of Medicine, The University of Tokyo, Tokyo, Japan; 17 Department of Neurosurgery and Neurooncology, National Cancer Center Hospital, Tokyo, Japan; 18 Department of Neurosurgery, Kurume University School of Medicine, Kurume, Fukuoka Japan; 19 Department of Neurosurgery, Faculty of Life Sciences, Kumamoto University Graduate School, Kumamoto, Japan; 20 Department of Neurosurgery, Yamagata University School of Medicine, Yamagata, Japan; 21 Department of Neurosurgery, Tohoku University Graduate School of Medicine, Sendai, Japan; 22 Department of Pediatric Neurosurgery, Osaka City General Hospital, Osaka, Japan; 23 Department of Neurosurgery, Kyorin University Faculty of Medicine, Tokyo, Japan; 24 Department of Neurosurgery, Graduate School of Medical Sciences, Kanazawa University, Kanazawa, Japan; 25 Department of Pathology, Kurume University, Kurume, Fukuoka Japan; 26 Department of Neurosurgery, Fujita Health University, Toyoake, Aichi Japan; 27 Department of Neurosurgery, Showa University School of Medicine, Tokyo, Japan; 28 Department of Neurological Surgery, Dentistry and Pharmaceutical Sciences, Okayama University Graduate School of Medicine, Okayama, Japan; 29 Department of Neurological Surgery, Wakayama Medical University, Wakayama, Japan; 30 Department of Neurosurgery, Nagano Children’s Hospital, Nagano, Japan; 31 Department of Neurosurgery, Osaka University Graduate School of Medicine, Osaka, Japan; 32 Department of Neurosurgery, Hyogo Prefectural Kobe Children’s Hospital, Kobe, Hyogo Japan; 33 Department of Pathology, Hyogo Prefectural Kobe Children’s Hospital, Kobe, Hyogo Japan; 34 Department of Neurosurgery, Tazuke Kofukai Foundation, Medical Research Institute and Kitano Hospital, Osaka, Japan; 35 Department of Neurosurgery, Shizuoka Children’s Hospital, Shizuoka, Japan; 36 Department of Pediatrics, The Jikei University School of Medicine, Tokyo, Japan; 37 Department of Neurosurgery, University of Occupational and Environmental Health, Kitakyushu, Fukuoka Japan; 38 Department of Neurosurgery, Kanazawa Medical University, Kanazawa, Japan; 39 Department of Neurosurgery, Toyama University Hospital, Toyama, Japan; 40 Department of Neurosurgery, Faculty of Medicine, Kinki University, Osaka, Japan; 41 Department of Neurosurgery, Kansai Medical University, Hirakata, Osaka Japan; 42 Department of Neurosurgery, Hokkaido Medical Center for Child Health and Rehabilitation, Sapporo, Japan; 43 Department of Pathology and Applied Neurobiology, Graduate School of Medical Science, Kyoto Prefectural University of Medicine, Kyoto, Japan; 44 Department of Pathology, Osaka National Hospital, National Hospital Organization, Osaka, Japan; 45 Developmental & Stem Cell Biology Program, The Hospital for Sick Children, Toronto, ON Canada; 46 Division of Neurosurgery, Hospital for Sick Children, Toronto, ON Canada; 47 Department of Neurosurgery, Teikyo University Hospital, Mizonokuchi, Kanagawa Japan; 48 Department of Pediatric Neurosurgery, Takatsuki General Hospital, Takatsuki, Osaka Japan; 49 Department of Neurosurgery, Juntendo University, Tokyo, Japan

**Keywords:** Ependymal tumors, Fusion gene, Gene rearrangement, Molecular classification

## Abstract

Extensive molecular analyses of ependymal tumors have revealed that supratentorial and posterior fossa ependymomas have distinct molecular profiles and are likely to be different diseases. The presence of *C11orf95-RELA* fusion genes in a subset of supratentorial ependymomas (ST-EPN) indicated the existence of molecular subgroups. However, the pathogenesis of *RELA* fusion-negative ependymomas remains elusive. To investigate the molecular pathogenesis of these tumors and validate the molecular classification of ependymal tumors, we conducted thorough molecular analyses of 113 locally diagnosed ependymal tumors from 107 patients in the Japan Pediatric Molecular Neuro-Oncology Group. All tumors were histopathologically reviewed and 12 tumors were re-classified as non-ependymomas. A combination of RT-PCR, FISH, and RNA sequencing identified *RELA* fusion in 19 of 29 histologically verified ST-EPN cases, whereas another case was diagnosed as ependymoma *RELA* fusion-positive via the methylation classifier (68.9%). Among the 9 *RELA* fusion-negative ST-EPN cases, either the *YAP1* fusion, *BCOR* tandem duplication, *EP300-BCORL1* fusion, or *FOXO1-STK24* fusion was detected in single cases. Methylation classification did not identify a consistent molecular class within this group. Genome-wide methylation profiling successfully sub-classified posterior fossa ependymoma (PF-EPN) into PF-EPN-A (PFA) and PF-EPN-B (PFB). A multivariate analysis using Cox regression confirmed that PFA was the sole molecular marker which was independently associated with patient survival. A clinically applicable pyrosequencing assay was developed to determine the PFB subgroup with 100% specificity using the methylation status of 3 genes, *CRIP1*, *DRD4* and *LBX2*. Our results emphasized the significance of molecular classification in the diagnosis of ependymomas. *RELA* fusion-negative ST-EPN appear to be a heterogeneous group of tumors that do not fall into any of the existing molecular subgroups and are unlikely to form a single category.

**Electronic supplementary material:**

The online version of this article (10.1186/s40478-018-0630-1) contains supplementary material, which is available to authorized users.

## Introduction

Ependymal tumors are classified into four histopathological subtypes including subependymoma (grade I), myxopapillary ependymoma (grade I), ependymoma (grade II), ependymoma, *RELA* fusion-positive (grade II or III), and anaplastic ependymoma (grade III) according to the World Health Organization (WHO) Classification of Tumours of the Central Nervous System [8]. Each of the latter two, which are clinically malignant, is defined as “A circumscribed glioma composed of uniform small cells with round nuclei in a fibrillary matrix and characterized by perivascular anucleate zones (pseudorosettes) with ependymal rosettes also found in about one quarter of cases (ependymoma), a high nuclear-to-cytoplasmic ratio, and a high mitotic count (anaplastic ependymoma)” [8]. Ependymal tumors, which may arise from any part of the neuroaxis, are identified as supratentorial (ST), posterior fossa (PF) or spinal (SP) ependymomas (EPNs). Malignancy grading for EPN and anaplastic EPN is often inconsistent, and the clinical significance of EPN pathological grading is controversial [20, 25, 28, 32]. Regardless of location, standard treatment for these tumors involves maximal safe surgical resection followed by local radiation therapy [29]. Incomplete resection or recurrence predicts a dismal prognosis. At present, there are no reports of chemotherapeutic agents with proven efficacy against these tumors [11, 35]. Therefore, further clarification of molecular mechanisms underlying the genesis of EPN, as well as development of new treatments for these tumors may be essential.

A series of extensive molecular analyses has demonstrated that supratentorial and posterior fossa EPNs may have distinct molecular profiles and are most likely separate diseases [19, 25, 27, 29, 37]. Recently, a consensus scheme for the molecular classification of EPNs based on these studies has been proposed [25]. ST-EPNs were segregated into two molecular subgroups denoted as ST-EPN-*RELA* and ST-EPN-*YAP1*. ST-EPN-*RELA* tumors, which are characterized by the presence of various types of *C11orf95-RELA* fusion genes, account for approximately 70% of ST-EPNs [27]. Some *C11orf95-RELA* fusion genes have been experimentally demonstrated as oncogenic. ST-EPN-*YAP1* subgroup is characterized by the *YAP1*. ST-EPN-*YAP1* fusion gene, which is much less common than ST-EPN-*RELA.* The oncogenic potential of most *YAP1* fusions remain to be determined. However, fusion genes are often intimately implicated in tumorigenesis. Therefore, fusion genes that are highly specific to ST-EPN are likely to be promising therapeutic targets for these particular tumors. However, diagnosis and clinical significance of the *C11orf95*-*RELA-* or *YAP1-*fusion negative ST-EPNs remains controversial. Whether these tumors harbor an unidentified driver event or belong to an entity that is entirely different from EPN, needs to be determined. Thus, a detailed investigation of the molecular profiles of these tumors was felt to be imperative.

According to the methylation pattern, PF-EPNs are segregated into two main molecular subgroups termed PF-EPN-A (PFA) and PF-EPN-B (PFB) [19, 25]. PFA subgroup tumors are characterized by an increased DNA methylation pattern in the CpG islands, which is different from that seen in PFB ependymomas. PFA patients are mostly infants or young children associated with a poor prognosis whereas PFB patients are older with better prognoses [19, 25]. No recurrent driver mutation, identifiable as a therapeutic target or diagnostic marker, has been found in either subgroup. Because the presence of biologically distinct subgroups within PF-EPN has significant clinical implications, validation of these sub groups as well as development of robust diagnostic tools for these groups are deemed essential.

For the purpose of molecular and clinical characterization of ependymal tumors and identification of therapeutic targets, we performed molecular analyses on a considerable series of ependymal tumors collected via the Japan Pediatric Molecular Neuro-oncology Group (JPMNG). These cases were examined using detailed clinical information and centrally reviewed histopathology. We confirmed that *RELA* fusion is a highly specific diagnostic marker for ST-EPN, and that methylation-based classification of PF-EPN is robust and may serve as an independent prognostic marker. We found that a considerable proportion of histopathologically diagnosed ST-EPN does not contain the *RELA* fusion, and that the molecular pathogenesis of these tumors may be complex.

## Materials & methods

### Tumor material

A total of 113 locally diagnosed ependymal tumors collected from 107 patients through JPMNG were examined in this study (Table 1). Among these, 38 were supratentorial, 63 were posterior fossa and 12 were spinal tumors. In all cases, a consensus diagnosis was made by three neuropathologists (A.S., T.H., and J.H.) following an extensive microscopic review of slides stained with hematoxylin, eosin and other immunohistochemistry procedures. In addition, 69 PF-EPN samples from the Hospital for Sick Children, Toronto, Canada, were included for validation of a pyrosequencing assay developed for molecular classification of PF-EPN (see below). This study was approved by the ethics committees of the National Cancer Center as well as the respective local institutional review boards.

**Table 1:** Patient characteristics

Total number of enrolled patients		107
Male:female ratio		55: 51 (Unknown, 1)
Observation period (median, range)		49 months (0–219)
Age (median, range)	10 years, (0–76 years)	
	< 3	22
	3–18	42
	18<	42
	Unknown	1
Extent of resection of the primary tumors	Total resection	51
	Partial resection/biopsy	47
	Unknown	9
Adjuvant therapy for the primary tumors	Radiation therapy (RTx)	32
	Chemotherapy (CTx)	13
	RTx + CTx	22
	No adjuvant therapy	29
	No data	11
Total number of enrolled samples		113
Tumor locations	Supratentorial	38
	Posterior fossa	63
	Spine	12
Time of surgery for the samples	Primary	91
	Recurrent	18
	Unknown	4
Pathological diagnosis (Institutional diagnosis)	Grade 1	2
	Grade 2	46
	Grade 3	60
	No data of grading	5
Pathological diagnosis (Central diagnosis)	Grade 1	1
	Grade 2	33
	Grade 3	67
	Other diagnoses	12
Molecular status	RELA fusion positive	20
	YAP1 fusion positive	1
	PFA	45
	PFB	15

### DNA/RNA extraction, cDNA synthesis, and bisulfite modification of DNA

DNA and RNA were extracted from the tumor samples, using a DNeasy Blood & Tissue kit (Qiagen, Tokyo, Japan) and Qiagen miRNeasy Mini Kit, respectively. First strand cDNA was synthesized using a SuperScript III Reverse Transcriptase kit (Life Technologies, Tokyo, Japan), according to the manufacturer’s instructions. Bisulfite modification of DNA was performed using an EZ Methylation DNA Kit (Zymo Research, CA, USA).

### Genome-wide DNA methylation analysis

Genome-wide DNA methylation analysis was performed using an Infinium HumanMethylation450 BeadChip array (Illumina, San Diego, CA, USA, hereafter 450 array) which includes 485,512 CpG sites for analysis, as described previously [11, 38]. For the methylation array, 500 ng of DNA extracted from fresh frozen specimens and 100 ng DNA from formalin-fixed paraffin-embedded (FFPE) specimens, repaired using an Infinium HD FFPE Restore Kit (Illumina, San Diego, CA, USA), was used. We removed 11,551 probes mapped on sex chromosomes and employed the remaining 473,961 probes for analysis. The methylation level of each CpG site was expressed using beta-values, ranging from 0 (unmethylated) to 1 (fully methylated). A total of 3086 probes showing a high standard deviation (SD > 0.25) on CpG islands, were selected for PF-EPN classification. Unsupervised hierarchical clustering was performed using R software (version 3.0.1), as described previously [15]. Methylation data of 48 EPNs (GSE42752) and 6 normal cerebellums (GSE44684) [17, 19, 33] were obtained from the Gene Expression Omnibus database (http://www.ncbi.nlm.nih.gov/geo/) for comparison with our data. The methylation profiling classifier developed by the German Cancer Research Center (DKFZ)/University Hospital Heidelberg/German Consortium for Translational Cancer Research (DKTK) (the DKFZ classifier, molecularneuropathology.org) was used via their website to assign subtype scores for each tumor [4].

### Copy number analysis

Copy number alterations were evaluated using signal data from the methylation array. Following an evaluation of methylated and unmethylated signals in the six normal cerebellum samples, probes showing high variability were excluded from the analysis [17]. Probes outside the 0.05 and 0.95 quantiles of median summed values, as well as probes over the 0.8 quantile of the median absolute deviation were excluded. Sample to median Log2-ratios of control samples at each probe were calculated and normalized against the median log2-ratio. Copy number data were obtained using the DKFZ classifier.

### PCR, RT-PCR, and sanger sequencing

PCR and RT-PCR were performed using an AmpliTaq Gold 360 kit (Applied Biosystems, Foster City, CA, USA). Following purification with ExoSAP-IT (Affymetrix USB, Cleveland, OH, USA), Sanger sequencing was performed using a BigDye Terminator v1.1 Cycle Sequencing Kit (Applied Biosystems, Foster City, CA, USA) for screening *TERT* C228T and C250T mutations, and a BigDye Terminator v3.1 Cycle Sequencing Kit (Applied Biosystems, Foster City, CA, USA) for screening other genes on an auto sequencer (3130xl Genetic Analyzer, Applied Biosystems, Foster City, CA) in the sequencing analysis. The primer sequences are shown (Additional file 1 Table S1).

### Fluorescence in situ hybridization (FISH)

Break-apart FISH was used to detect *RELA* or *YAP1* fusion. FISH was performed on 4–5 μm sections of each FFPE specimen. FISH probes were derived from the following BAC clones: for *RELA* fusion detection, RP11-472D15 and RP11-58D3 probes were labeled with spectrum orange, whereas RP11-692F22 and CTD-2121 M3 probes were labeled with spectrum green; for *YAP1* fusion detection, RP11-732A21 and RP11-640G3 were labeled with spectrum orange and RP11-1082I3 and RP11-315O6 with spectrum green. Briefly, BAC DNA from an overnight culture of the corresponding BAC clones was purified using a GenElute Plasmid Miniprep Kit (Sigma-Aldrich, Tokyo, Japan), amplified using a Templiphi Amplification Kit (GE Healthcare, Tokyo, Japan) and labeled using a Nick Translation Kit (Abbott Molecular, Abbott Park, IL) with appropriate dye-coupled dUTP, as per manufacturer’s instructions. Fluorescence in situ hybridization was performed as previously described [22]. Scoring of FISH results was performed using a BZ-9000 fluorescence microscope (Keyence, Osaka, Japan) with appropriate filters at 1000× magnification. A tissue microarray containing a tumor with a known *YAP1* fusion, kindly provided by Dr. David Ellison from St. Jude Children’s Research Hospital, was used as a positive control.

### Expression analysis

mRNA expression levels were evaluated via real-time quantitative PCR (qPCR) using the LightCycler 480 SYBR Green I Master and the SYBR Green I (483–533 nm) detection format on a CFX96 (Bio-Rad Laboratories, Inc., Hercules, CA, USA), according to the manufacturer’s instructions. The primer pairs used to perform qPCR were as follows: *TERT -* forward primer (P570) located in exon 6 and reverse primer (P571) located in exon 7; and *EZH2* - forward primer (P563) located in exon 2 and reverse primer (P564) located in exon 3. The expression level of *H6PD*, determined via the primer pair, (P114) and (P115), was used as an internal reference for normalization. PCR conditions were as follows; 95 °C for 5 min, 45 cycles of 10 s at 95 °C each, 55 °C for 10 s and 72 °C for 10 s. A standard curve was generated using serially diluted cloned PCR products of both the internal reference and target genes. Expression was measured relative to the human total brain RNA (Clontech Laboratories, Mountain View, CA, USA). Primer sequences are described (Additional file 1 Table S1).

### Mutation analysis by pyrosequencing

Hot spot mutations of *IDH1* (R132), *IDH2* (R172), *BRAF* (V600E), *H3F3A* (K27 M, G34R), *FGFR1* (N546, N656) and *HIST1H3B* (K27 M) were evaluated via pyrosequencing. Methylation analysis of *TERT* promoter regions and/or 3 upstream transcription starting sites (UTSSs, R1, R2 and R3) were performed as reported previously [3, 5]. Primer sequences, analyzed sequences and the dispensation order are shown (Additional file 1 Table S1). Pyrosequencing was performed using the AQ assay of PyroMark Q96 (version 2.5.7) on a PyroMark ID pyrosequencer (Qiagen, Tokyo, Japan), according to the manufacturer’s instructions.

### PF-EPN subgroup prediction by pyrosequencing

A set of pyrosequencing assays was developed in order to sub-classify PF-EPN into PFA or PFB effectively. At first, 10 PFA and 10 PFB cases were investigated as a discovery set (Results and Additional file 2 Tables S2 and Additional file 3 Table S3). Highly methylated probes (mean beta-value ≥0.5) in PFA cases and hypomethylated probes (mean beta-value ≤0.2) in PFB cases, were selected from our discovery set and archival data set (*n* = 48) with an Infinium HumanMethylation450 BeadChip array [19]. Out of the 414,634 probes used, 13 probes covering 3 genes, *CRIP1*, *LBX2*, and *DRD4*, located in the autosomes were selectively identified, (Additional file 2 Table S2). Next, a set of pyrosequencing assays was designed to examine the methylation status of CpG sites, as well as their flanking CpG sites, targeted by the probes cg04411625 (*CRIP1*), cg03270710 (*LBX2*), cg20931042 and cg06825142 (*DRD4*). Primers and dispensation orders are listed (Additional file 1 Table S1). Methylation levels were measured using the CpG assay of PyroMark Q96 (see above). Mean methylation levels of all CpG sites included in each assay were used to represent the methylation status of each gene. Methylation levels at these CpG sites measured via pyrosequencing showed good concordance with those of the 450 K array (data not shown). The dataset containing 54 cases from the JPMNG cohort and 69 cases from the SickKids cohort was used in the training and validation processes for the prediction rule design, (Additional file 4 Figure S5a). The original dataset of JPMNG and SickKids was preprocessed via random sampling and divided on a 1:2 basis into a training dataset containing 41 cases (PFA: 30, PFB: 11) and a validation dataset containing 82 cases (PFA: 60, PFB: 22), while maintaining the PFA:PFB ratio. Statistical analysis and determination of the cutoff are described in Results.

### Whole transcriptome sequencing

The TruSeq RNA Sample Prep Kit (Illumina, CA, USA) was used to prepare RNA sequencing libraries from total RNA. Samples with an RNA integrity number of 6 or less were prepared using TruSeq Stranded Total RNA with Ribo-Zero Gold LT Sample Prep Kit (Illumina, CA, USA). The resultant libraries were subjected to paired-end sequencing of 75-bp reads on a HiSeq 2000 (Illumina, CA, USA). Fusion transcripts were detected using the TopHat-Fusion algorithm [14] (Additional file 5 Table S4). For expression analysis, we established “virtual” Agilent 8x60K array data from RNA sequencing data by counting the number of inserts expected to hybridize on probe positions of the array (VA). The data was normalized by the median number of inserts.

### Immunohistochemical analysis of H3K27me3

Of the 60 PF-EPNs that were subjected to consensus diagnosis and molecular subclassification into PFA or PFB, 44 cases were available for evaluation using immunohistochemistry. Four-micrometer-thick sections cut from blocks representing each tumor were deparaffinized. The preparations were autoclaved in citrate buffer (pH 6.0), and endogenous peroxidase activity was blocked with 3% hydrogen peroxide. The primary antibody used was the anti-H3K27me3 rabbit monoclonal antibody (C36B11, dilution 1:200; Cell Signaling Technology, Danvers, MA, USA). Slides were incubated for 1 h at room temperature with the primary antibody, and subsequently labeled by using the EnVision system (Dako, Glostrup, Denmark). Diaminobenzidine was used as the chromogen, and hematoxylin as the counterstain. The entire area of the stained slides was visually inspected, and the percentage of cells that lacked staining was assessed semi-quantitatively. Cases were categorized as showing intact expression when over 80% of tumor cells were intact for H3K27me3, or as showing reduced expression when 0–80% of tumor cells were labeled as intact [26]. Staining was deemed evaluable only if endothelial cells in the tumor tissue showed intact reactivity. Staining intensity was not used as a parameter for evaluation.

### Statistical analysis

Comparison between subgroups was performed using the Student’s *t*-test, Pearson’s chi-square test, Fisher’s exact test and the Wilcoxon rank-sum test. Overall survival (OS) was defined as the probability of survival, with death as the only event. Progression-free survival (PFS) was defined as the probability of being alive without a risk of progression or relapse. Survival curves were plotted using the Kaplan-Meier method. The log-rank test and Cox proportional hazards model were used to detect differences in survival between different groups of patients. Two-sided tests were used for all analyses, and the significance level was set at *P* < 0.05. JMP 10 (SAS Institute Inc., Cary, NC, USA) was used for all analyses.

## Results

### Central pathology review

A total of 113 locally diagnosed ependymomas (38 supratentorial, 63 posterior fossa and 12 spinal) analyzed in this study were subjected to a central review of histopathology (Table 1). Following this review, 1 myxopapillary EPN (grade I), 33 EPNs (grade II) and 67 anaplastic EPNs (grade III) were identified. Nine supratentorial and 3 posterior fossa tumors were re-classified as non-ependymal tumors. As a result, 29 supratentorial, 60 posterior fossa tumors (not including 3 re-reclassified posterior fossa tumors) and 12 spinal tumors were subjected to molecular analysis. Detailed results of histopathology related analyses will be published elsewhere (Sasaki, submitted).

### *C11orf95-RELA* fusion negative ST-EPNs are highly heterogeneous

**Fig. 1:**
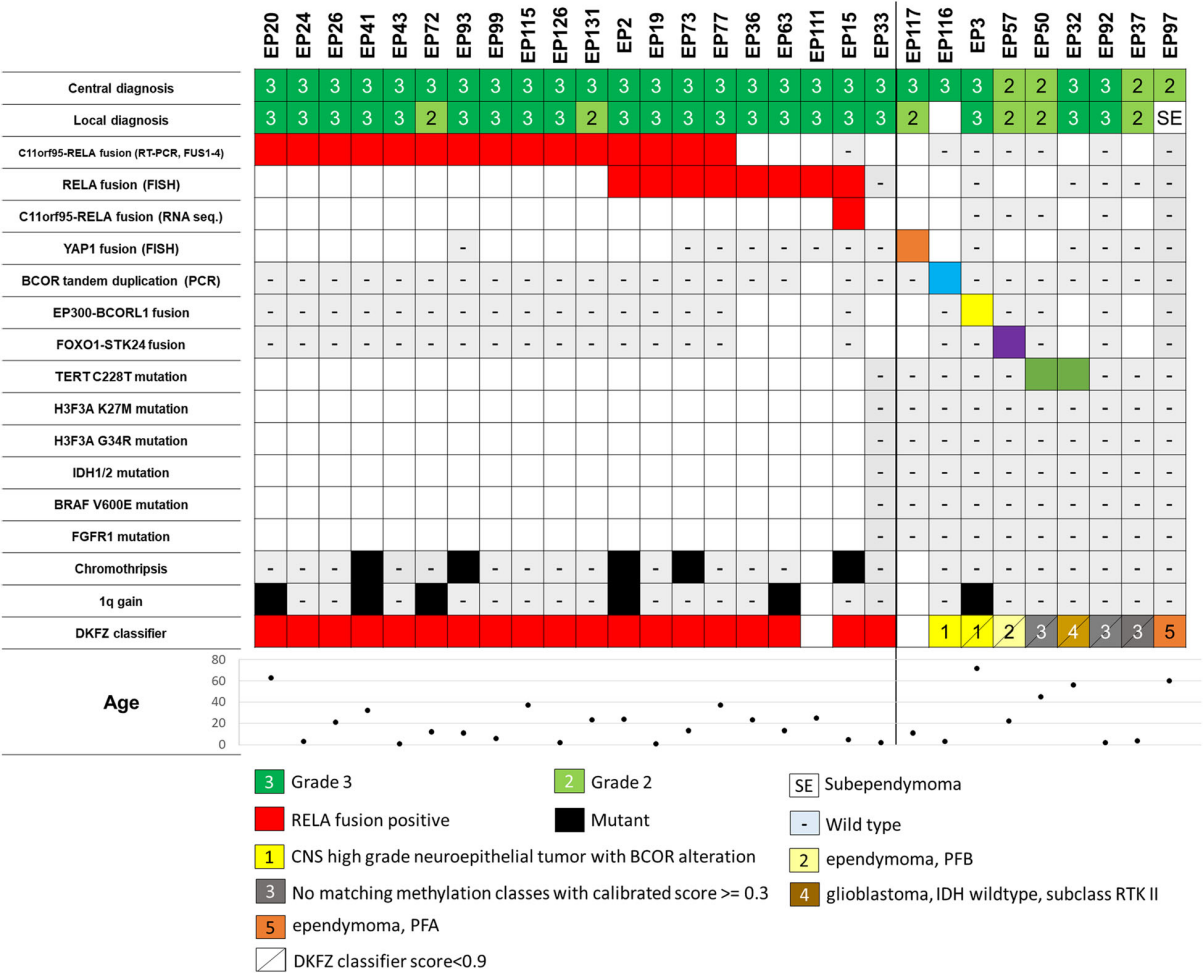
Clinical and genomic features of supratentorial ependymomas (ST-EPNs). Central and local histological diagnoses are indicated in the top column. All genotypes examined are shown (un-examined genotypes are left as blank). The results of the DKFZ classifier are shown in the bottom columns. Patients’ ages are indicated below the diagram. *C11orf95-RELA* fusions were detected among only ST-EPNs diagnosed by consensus diagnosis. ST tumors confirmed by consensus diagnosis without *C11orf95-RELA* fusions show various genetic alterations including *YAP1* fusion

It has been proposed that ST-EPNs may be divided into three molecular subgroups; ST-EPN-RELA (*RELA* fusion-positive), ST-EPN-YAP1 (*YAP1* fusion-positive) and ST-SE (subependymoma) [25, 27]. To validate the above molecular classification, we sought to identify fusions using a combination of RT-PCR, FISH, and/or RNA-sequencing analysis in ST-tumors (*n* = 38), including 9 tumors re-classified as non-ependymoma following a central review. *C11orf95-RELA* fusions were detected in 19 out of 29 ST-EPNs using RT-PCR and/or FISH (Fig. 1). All 19 *RELA*-fusion positive ST-EPNs were diagnosed as grade III after the central review. The RT-PCR used in this study detected 4 out of 7 *C11orf95-RELA* fusion transcripts reported so far [27]. A novel *C11orf95-RELA* fusion transcript, in which exon 2 of *C11orf95* was fused to exon9 of *RELA* in-frame, was detected via RNA sequencing in an ST-EPN with a RELA fusion identified by FISH, but not by RT-PCR (EP15, Additional file 6 Figure S1a). *C11orf95-RELA* fusion was not detected in any of the 9 tumors re-classified following central review. One case (EP33) in which *RELA* fusion was not detected by either RT-PCR or FISH was classified to be *RELA* fusion-positive using DKFZ classifier results (see below). A copy number analysis using the 450 K array showed a copy number loss of upstream exon 2 of *RELA,* the most common break point of the fusion gene. Immunohistochemical staining of L1 cell adhesion molecule (L1CAM) showed strong positivity. These findings corroborated the result of the classifier (Additional file 7 Figure S6). EP33 was not subjected to RNA sequencing due to insufficient amount of RNA, and likely to have RELA fusion other than those examined by RT-PCR. As a result, out of 29 histologically verified ST-EPNs a total of 20 (68.9%) were identified as *RELA* fusion-positive ependymomas. *YAP1* fusion was detected via FISH in only one grade III tumor (EP117, 1/29, 3.4%). In this case, the fusion could not be studied further due to insufficient specimens. Thus, 8 histologically verified ST-EPN had neither *RELA* nor *YAP1* fusions (Fig. 1). Chromothripsis, frequently found on chromosome 11 in ST-EPN is highly indicative of *C11orf95-RELA* fusion, and therefore considered to be one of its causal mechanisms [27]. Copy number analysis using 450 K array data indicated that of 20 ST-EPNs, 5 (25%, data not shown) exhibited a highly unstable chromosome 11, which is highly suggestive of chromothripsis. No evidence of chromothripsis was observed in ST-EPNs, in the absence of *RELA* fusion or non-ependymal tumors. There was no preferred age of onset, intracerebral location or tumor form (cystic or solid) in *C11orf95-RELA*-positive EPNs, as compared to *RELA*-negative tumors (Additional file 3 Table S3a).

Eight ST-EPNs negative for either *C11orf95-RELA* or *YAP1*-related fusion (4 grade II, and 4 grade III) were further investigated. Among those 8 cases, 3 demonstrated histological features of classic, Grade II ependymoma (EP97) or Grade III anaplastic ependymoma (EP3, EP32) whereas the remaining 5 exhibited evidence of ependymal differentiation as well as a variety of unusual features including astrocytic cells, tanycytic cells, vacuolated cells or microcysts (EP116, EP57, EP50, EP92, EP37, Additional file 8 Figure S7). RNA sequencing was performed in 5 tumors with sufficient amounts of RNA. We identified 2 novel in-frame fusion genes, *EP300-BCORL1* (exon 31-exon 4) in one grade III tumor (EP3) and *FOXO1-STK24* (exon 1-exon 3) in one grade II tumor (EP57) (Fig. 1, Additional file 6 Figure S1 and Additional file 9 Figure S4). EP57 with *FOXO1-STK24* fusion also exhibited copy number oscillations equivalent to chromothripsis in both chromosome 6 and 13, on which these genes are located (Additional file 6 Figure S1b). EP3 showed significantly higher expression of *BCORL1* than *RELA*-fusion cases and the other non-*RELA*-fusion cases (Additional file 10 Figure S8). EP57 showed increased expression of both FOXO1 and STK24 compared to other RELA-fusion or non-RELA fusion ST-EPNs. The histology of EP3 and EP57 is presented in Additional file 8 Figure S7. In addition, a *BCOR* tandem duplication recently reported in central nervous system-primitive neuroectodermal tumors (PNETs) was found in one case (EP116) with grade III ST-EPN [30] (data not shown). We further examined fusion-negative ST-EPNs by pyrosequencing for hot-spot mutations in *IDH1*, *IDH2*, *TERT*, *BRAF* V600E, *H3F3A*, *HIST1H3B* and *FGFR1* [1, 2] (Additional file 1 Table S1). *TERT* promoter mutations (C228T) were observed in one EPN grade II and one EPN grade III. The patients in these 2 cases were 45 and 56 years old. No mutations of the alterations examined were detected in the remaining cases.

In order to further validate our molecular classification, the DKFZ classifier was applied to all cases via the DKFZ molecular neuropathology website (see Materials and Methods), except EP111 (*RELA* fusion) and EP117 (*YAP1* fusion) which had insufficient material for an analysis to be performed. All *RELA*-positive ST-EPNs matched “methylation class ependymoma, *RELA* fusion” by the DKFZ classifier (score > = 0.90); (Fig. 1 and Additional file 3 Table S3). The *RELA*-negative ST-EPNs displayed variability in regard to methylation classes as follows: 3 (EP50, EP92, EP37) with no matching methylation classes (calibrated score > = 0.3; Fig. 1), 2 (EP116, EP3) with CNS high grade neuroepithelial tumors carrying the *BCOR* alteration (*BCOR* altered tumor), 1 (EP97) with ependymoma PFA, 1(EP57) with ependymoma PFB and 1 (EP32) with glioblastoma *IDH* wildtype subclass RTK II. Among the 3 cases with no matching methylation cases, 1 case carried a *TERT* promoter mutation and the other 2 cases exhibited no alterations via pyrosequencing of selected genes or RNA sequencing. Of the 2 tumors carrying *BCOR/BCORL1* alterations, EP116 with a verified *BCOR* tandem duplication was classified as “CNS high grade neuroepithelial tumor with *BCOR* alteration” (score = 0.99), whereas EP3 with the *EP300-BCORL1* fusion with no match, was classified as “CNS high grade neuroepithelial tumor with *BCOR* alteration” with a low score (0.44). EP57, with the *FOXO1-STK24* fusion, was classified as ependymoma PFB with a low score (0.44). EP57 was a left occipital lobe tumor extending to the lateral ventricular wall, which was completely removed by surgery. EP97 was located in the right lateral ventricle, which was partially removed. Notably, of the ST-tumors re-classified as non-ependymomas by the central histology review, one tumor re-diagnosed as glioblastoma carried *H3F3A* K27 M, and another re-diagnosed glioblastoma carried G34R (Additional file 3 Table S3). A *BCOR* tandem duplication was found in a high grade malignant tumor, not otherwise specified. These genotypes were matched with the DKFZ methylation classes with high scores.

### PF-EPNs are subclassified into PFA and PFB by methylation profile

**Fig. 2:**
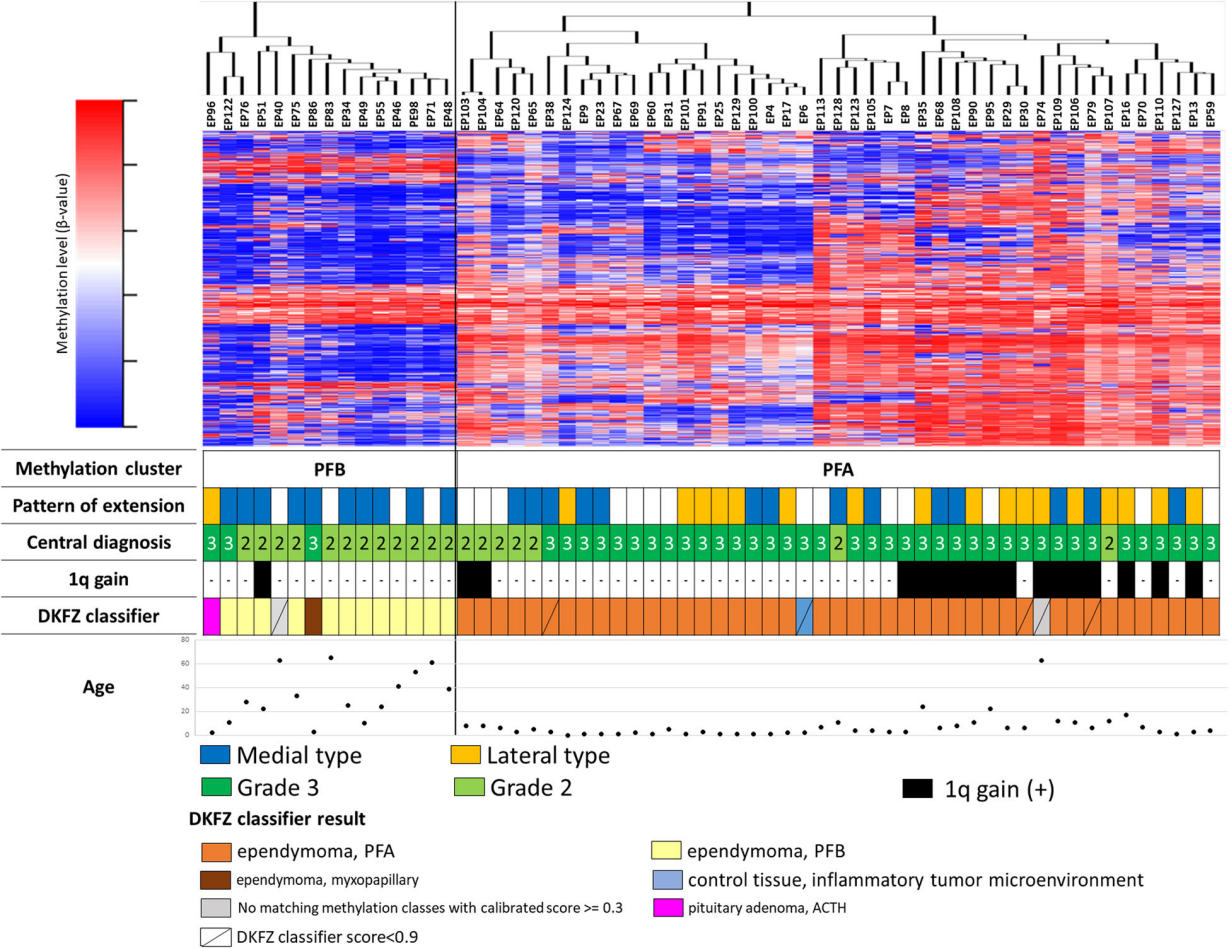
Classification of posterior fossa ependymomas (PF-EPNs) using genome-wide methylation profiling. A heatmap analyzed by 3086 probes which showed high standard deviations (SD > 0.25) on CpG islands for unsupervised hierarchical clustering of 60 centrally-diagnosed posterior fossa ependymomas shows that the tumors are divided into two clusters as PFA and PFB. The following information is indicated below the heatmap: tumor location, a pattern of PF tumor extension, pathological grading, the presence of 1q gain, age at onset, and the DKFZ classifier results

Although no recurrent genetic alterations have been identified in PF-EPNs, it was proposed that the PF-EPNs be segregated into two subgroups; PFA and PFB [19, 25]. To validate methylation-based classification, we investigated genome-wide methylation status of 60 PF-EPNs together with clinical information. Our 450 K array analysis segregated these PF-EPNs into two subgroups with distinct methylation profiles (Fig. 2). When our PF-EPNs were combined with a published PF-EPN dataset, the Toronto cohort (Material & Methods), and analyzed, each of these two subgroups was clustered with published PFA or PFB, indicating that the 450 K array analysis was robust and accurately identified these two subgroups (data not shown). Our PFAs were generally matched with the DKFZ classifier results, although with lower scores in some cases, except 2 PFAs for which no match could be found. These 2 could not be even assigned as normal tissue. Posterior fossa PFB were mostly correctly diagnosed by the classifier as well, except for 3 tumors that were classified as a pituitary adenoma (EP96), ependymoma and a myxopapillary (EP86) and no matching class (EP40). When PF-EPN and SP-EPN were collectively analyzed, all but one spinal tumors were segregated with PFB (Additional file 11 Figure S2). Nine SP-EPN were classified by the DKFZ classifier as spinal ependymomas, one as an adult plexus tumor, one as a pituitary adenoma and one with no matching class.

When molecular classification results were compared with clinical characteristics of intracranial PF-EPNs, excluding spinal EPN, PFA tumors (*n* = 45) occurred predominantly in younger patients (*p* < 0.001) and were laterally rather than medially located (*p* = 0.028) compared to PFB tumors (Additional file 3 Figure S3a, b). The great majority of PFAs were grade III while most PFBs were grade II (*p* < 0.001, Additional file 3 Figure S3c). There was no difference in the resection rate between PFA and PFB (Additional file 3 Figure S3d). All 1q gain but one occurred in PFA (Fig. 2). PFA with 1q gain was observed in older patients (*p* < 0.001) and tended to develop spinal dissemination at onset (*p* = 0.09) as compared to PFA without 1q gain (Additional file 3 Figure S3e, g).

### PFA is the most significant prognostic factor in all EPNs

We evaluated the prognosis prediction efficacy of molecular markers as well as clinical/pathological factors potentially associated with the survival of EPN patients. Only primary tumors were included in the survival analysis. High levels of *EZH2* protein, *TERT* mRNA expression and hypermethylation of *TERT* upstream transcription starting sites (UTSSs) have previously been reported to be correlated with negative prognoses for EPN patients [5, 13, 16, 18, 21]. We investigated the status of these genes in the EPN cohort. Among the 4 molecular groups, ST-EPNs showed the highest *EZH2* and *TERT* mRNA expression (Additional file 5 Figure S4a and S4b). Notably, *TERT* mRNA expression was 10 to 100 times higher in the *C11orf95-RELA* fusion-positive EPNs compared to all other EPN groups and even adult GBMs with the *TERT* promoter mutation (Additional file 5 Figure S4b). *TERT* UTSSs were highly methylated in the *C11orf95-RELA* fusion-positive ST-EPNs with high *TERT* mRNA expression (Additional file 5 Figure S4c).

A univariate analysis of all above data was performed to examine the efficacy of predicting prognosis of incomplete resection, WHO grade III, *C11orf95-RELA* fusion, PFA, 1q gain, high *EZH2* expression, high *TERT* expression, and high *TERT* UTSS methylation status in all EPN patients whose clinical and molecular information was available (30 ST, 67 PF + SP). High expression/methylation in *EZH2/TERT* was determined to be above the respective median value. The results showed that WHO grade III (*p* = 0.006), PFA (*p* = 0.0004) and 1q gain (*p* = 0.0003) were significantly associated with progression-free survival (PFS) (Table 2). WHO grade III (*p* = 0.001) and PFA (*p* = 0.0004) were significantly associated with shorter overall survival (OS). *C11orf95-RELA* fusion, high *EZH2* expression, high *TERT* expression, or high *TERT* UTSS methylation were not associated with survival. Incomplete resection was not significantly associated with survival, even though there was a tendency towards predicting shorter survival among all ependymomas or in PFA or PFB (Additional file 15 Figure S9; Discussion). Multivariate analysis using Cox regression of the same set of clinical factors and molecular markers showed that PFA was the only factor that was independently associated with PFS and OS among all EPNs (*p* = 0.002 for PFS; *p* = 0.01 for OS; Table 2). Univariate and multivariate analyses of the tumors in each location are described (Additional file 12 Table S6).

**Table 2:** Univariate and Multivariate analysis of progression free survival (PFS) and Overall survival (OS) among all tumors

Variable	Hazard ratio (HR)	95% confidence interval for HR	*p*-value
Univariate analysis of PFS among all tumors			
Incomplete resection	1.66	0.30–1.17	0.13
WHO grade3	2.91	1.34–7.26	0.0057
C11orf95-RELA fusion	0.59	0.29–1.50	0.29
PFA	3.30	1.69–6.72	0.0004
1q gain	3.21	1.50–6.48	0.0037
EZH2 high expression	1.29	0.63–2.67	0.49
TERT high expression	1.18	0.58–2.42	0.65
TERT UTSS high methylation	1.42	0.72–2.96	0.32
Local radiation therapy> = 50Gy	0.73	0.37–1.46	0.37
Chemotherapy	1.48	0.74–2.90	0.26
Multivariate analysis of PFS among all tumors			
WHO grade3	1.33	0.53–3.66	0.55
PFA	3.09	1.48–6.81	0.0024
1q gain	2.79	1.25–5.99	0.014
Univariate analysis of OS among all tumors			
Incomplete resection	2.22	0.17–1.09	0.077
WHO grade3	6.31	1.84–39.6	0.0017
C11orf95-RELA fusion	0.46	0.07–1.61	0.25
PFA	5.47	2.16–16.7	0.0002
1q gain	1.57	0.51–3.99	0.40
EZH2 high expression	1.21	0.46–3.21	0.70
TERT high expression	0.76	0.29–1.94	0.57
TERT UTSS high methylation	1.90	0.78–5.28	0.16
Local radiation therapy> = 50Gy	1.22	0.48–3.31	0.68
Chemotherapy	1.86	0.74–4.62	0.18
Multivariate analysis of OS among all tumors			
WHO grade3	3.49	0.91–23.05	0.07
PFA	3.54	1.33–11.4	0.01

**Fig. 3:**
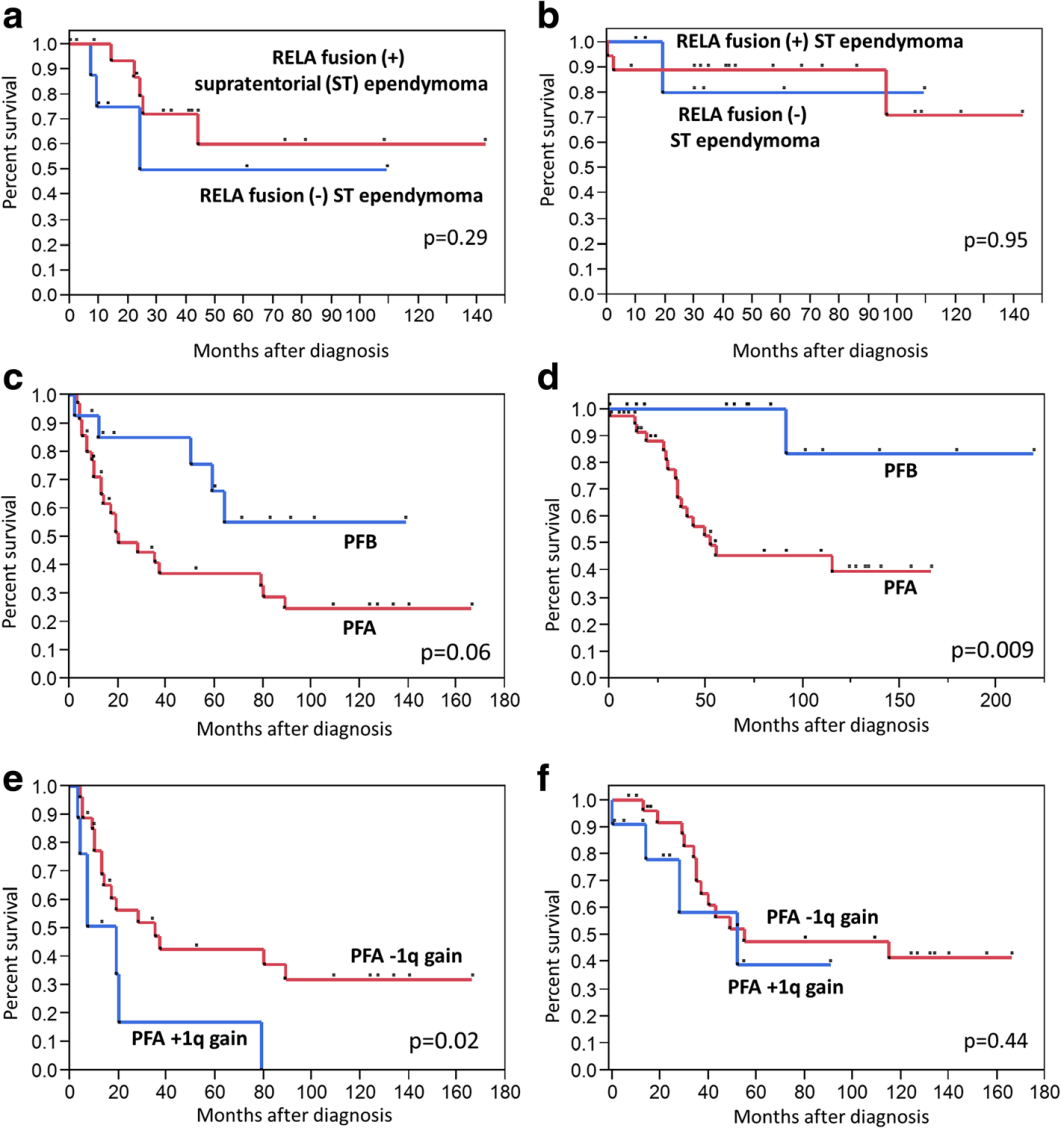
Survival of ST-EPNs stratified according to the presence of *C11orf95-RELA* fusions. **a** Progression-free survival (PFS), **b** overall survival (OS). There was no survival difference between the two groups. (c-d) PFS (**c**) and OS (**d**) of PFA and PFB. Significant differences in OS (*p* = 0.009) were observed between PFA and PFB patients. (**e**-**f**) PFS (**e**) and OS (**f**) of PFA with or without 1q gain. A significant difference in PFS (*p* = 0.02) but not in OS was observed between them

Finally, the efficacy of molecular classification in predicting prognosis in EPN patients was investigated separately for ST- or PF-EPN patients. No significant difference in survival was observed between the *C11orf95*-*RELA* fusion positive and negative ST-EPNs (Fig. 3a, b). Survival data was not available for EP116. Patients with PFA tumors showed a tendency towards shorter progression free survival (*p* = 0.06, Fig. 3c) and significantly shorter overall survival (*p* = 0.009, Fig. 3d) than those with PFB tumors. Among patients with PFA, those with 1q gain showed significantly shorter PFS than those without 1q gain (*p* = 0.02; Fig. 3e). There was no significant difference in overall survival between PFA patients with and without 1q gain (*p* = 0.44, Fig. 3f).

### PF-EPN subgroup prediction by methylation thresholds of the three genes

**Fig. 4:**
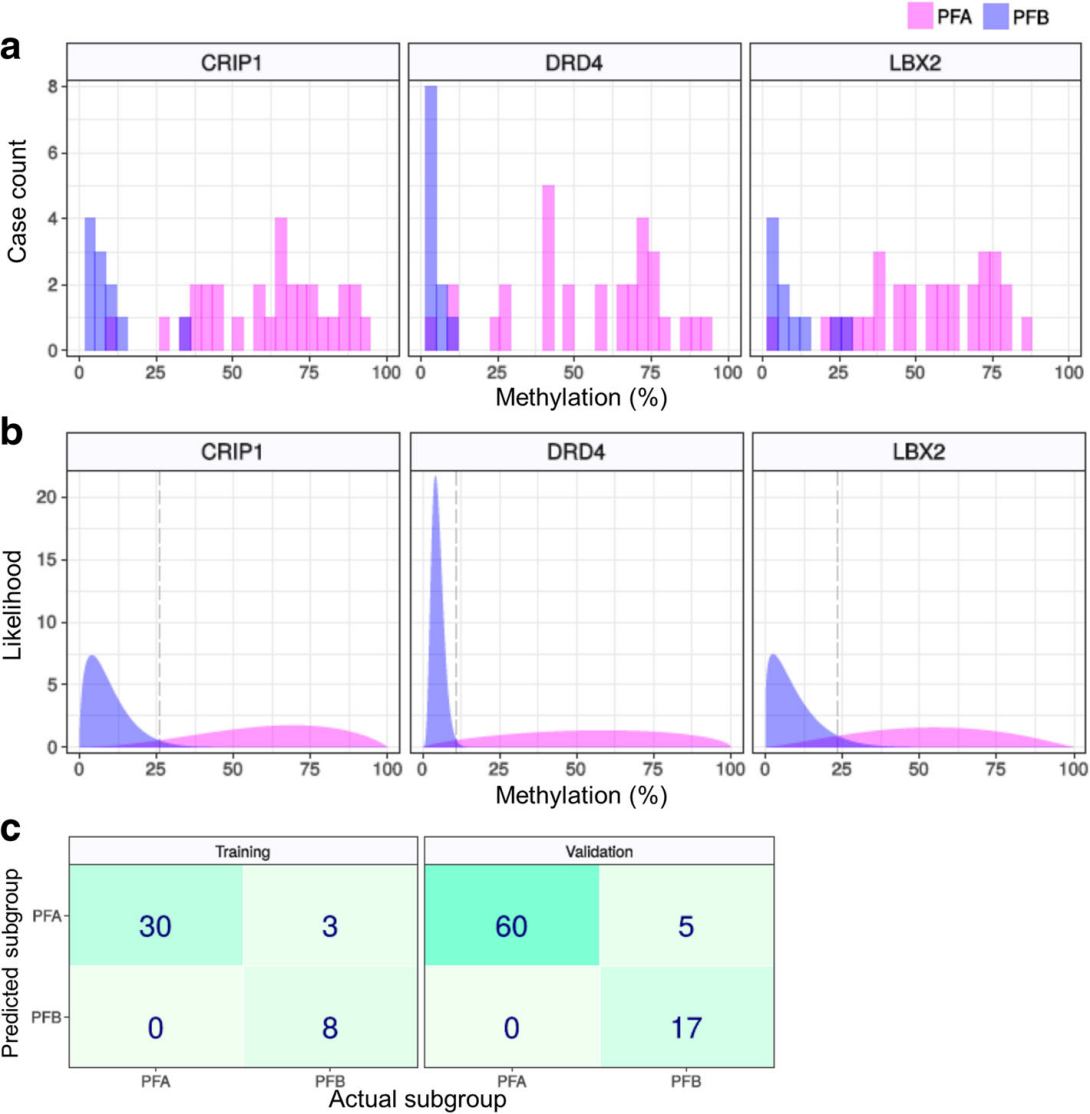
Prediction of PF-EPN subgroups using methylation thresholds of *CRIP1*, *DRD4*, and *LBX2*. **a** Methylation percentages for the three genes in the training dataset. **b** Likelihoods for each subgroup calculated by presuming beta distribution. The long-dashed lines denote thresholds determined by likelihood ratios. **c** Confusion matrices of prediction with training and validation datasets, according to the rule that classifies a case as PFB if all three genes suggest PFB

**Fig. 5:**
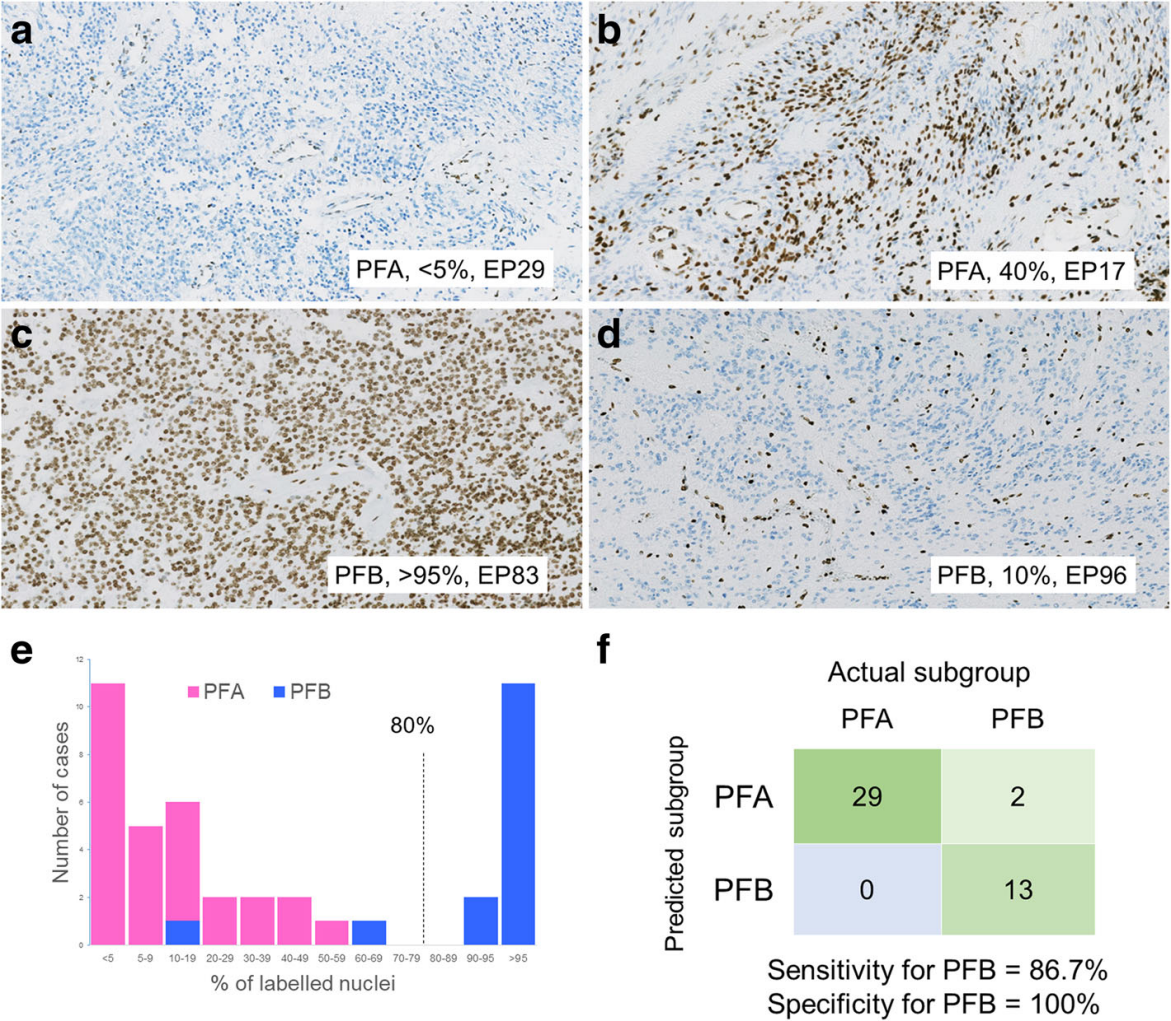
Immunohistochemistry for H3K27me3 in PFA and PFB tumors. All PFA tumors demonstrated reduced H3K27me3 expression (80% or less). Approximately 38% of them showed reactivity in less than 5% of tumor cells (**a**, **e**), and the remaining cases showed labeling in 5–50% of tumor cells (**b**, **e**). In contrast, most PFB tumors retained intact H3K27me3 expression (> 80% labeled nuclei) (**c**, **e**). A few PFB tumors, however, showed labeling in 10–60% of cells, which were categorized as reduced expression of H3K27me3 (**d**, **e**). **e**, a histogram of the percentage of labeled nuclei in PFA and PFB tumors. **f**, a confusion matrix for actual and predicted subgroup by H3K27me3 immunohistochemistry when PFB was defined as intact H3K27me3 expression and PFA as reduced expression

Next, we developed a PF-EPN subgroup prediction assay using DNA methylation percentage thresholds for *CRIP1*, *DRD4*, and *LBX2*. R version 3.4.3 (R Foundation for Statistical Computing, Vienna, Austria) was used for all analyses. In the training process, likelihoods for each gene and subgroup were calculated using the training dataset assuming beta distribution, and thresholds for each gene were determined to be 25, 11, and 23%, respectively based on the likelihood ratio (Figs. 4a, b). In the validation process, prediction results for each gene were obtained from both training and validation datasets, and three rule candidates were validated by the results (Additional file 3 Table S3, Additional file 13 Table S5; Additional file 4 Figure S5b). The first of the three candidates classified a case as PFB if all of genes suggested PFB, the second classified a case as PFB if a majority of genes suggested PFB and the last classified a case as PFB if any gene suggested PFB. All candidates classified a case as PFA if they did not classify it as PFB. Finally, a rule candidate that classified a case as PFB if all three genes suggested PFB was defined as the prediction rule, which showed highest specificities for PFB in the both datasets (training: 1.0, validation: 1.0) (Fig. 5c; Additional file 13 Table S5). Our results thus indicated that the methylation status of these three genes may predict the molecular subgroup of PF-EPNs with 100% specificity for PFB.

### Immunohistochemical analysis of H3K27me3 for PF-EPNs

Finally, we determined the relationship between H3K27me3 immunostaining and molecular subclassification of PF-EPNs based on the 450 K array. The 44 JPMNG cases whose H3K27me3 immunostaining results were available were classified either as intact or reduced expression by two pathologists (A.Y. and K.S). These studies showed that all 29 (100%) PFA cases showed reduced expression of H3K27me3, while 13 out of 15 (86.7%) and 2 out of 15 (13.3%) of PFB cases showed intact and reduced H3K27me3 expression, respectively. Among 29 PFA cases, which showed reduced H3K27me3 immunoreactivity, less than 5% of tumor cells in 11 cases showed H3K27me3 expression (Fig. 5a) and 5–50% tumor cells in 18 cases showed H3K27me3 labeling (Fig. 5b). In contrast, among the 15 PFB cases, 13 retained intact H3K27me3 expression (Fig. 5c), whereas 2 showed labeling, categorized as reduced expression, in 10–60% of cells (Fig. 5d). Thus, when a cutoff of 80% labeled nuclei was used as suggested by Panwalkar et al. [26], intact (> 80%) H3K27me3 expression predicted PFB with 100% specificity (Fig. 5e,f).

## Discussion

Molecular classification is essential for integrated diagnosis of central nervous system tumors in modern diagnostic pathology. However, using such classification in ependymomas is challenging due to the very limited number of available markers. In this study, the proposed molecular classification of supratentorial and posterior fossa ependymomas was extensively investigated in an independent set of 113 locally diagnosed ependymal tumors in Japan.

Our study confirmed that *C11orf95-RELA* fusion is a unique genetic feature of ST-EPN and that its presence is consistent with histopathological diagnosis. On the other hand, pathogenesis of ST-EPN in the absence of *C11orf95-RELA* fusion remains unresolved. There were 9 *C11orf95-RELA* fusion-negative ST-EPNs including 4 ependymoma grade IIs and 5 anaplastic ependymoma grade IIIs. Thus, those tumors were histologically confirmed as ependymoma, by definition. None of these were diagnosed as subependymoma following central review, where only a single case of *YAP1* fusion was identified by FISH (EP117). Instead, 2 novel fusion genes, *EP300-BCORL1* and *FOXO1-STK24,* were detected in single cases (EP3 and EP57).

*EP300* (E1A binding protein p300, located at 22q13) is a transcriptional coactivator that binds to a variety of transcription factors and bridges them to basal transcription machinery, and additionally functions as histone acetyltransferase that relaxes chromatin structure [5]. *BCORL1* (BCL6 Corepressor Like 1, located Xq26.1) is a transcriptional corepressor that interacts with histone deacetylases to repress transcription of genes such as E-cadherin [23]. The *EP300-BCORL1* fusion found in EP3 retained nearly all functional domains of both genes, but *BCORL1* expression was significantly increased in EP3 (Additional file 10 Figure S8). Interestingly, 2 ossifying fibromyxoid tumors with a *CREBBP-BCORL1* fusion have been reported [12]. *CREBBP (CBP)* is a paralog of *EP300* and as their functions mostly overlap they are often collectively described as *CBP/EP300*) [5]. *BCORL1* was overexpressed in *CREBBP1-BCORL1* fusion-positive tumors, suggesting that activation of *BCORL1* may be a consequence of *CREBBP1-BCORL1*. Thus, it is likely that *BCORL1* activation, a consequence of *EP300-BCORL1*, may lead to deregulation of chromatin remodeling through recruitment of histone deacetylase. In addition, *BCOR* (BCL6 corepressor) internal tandem duplication (ITD), which acts as an activating oncogene [34], was also found in a single ST-EPN in our cohort (EP116). The DKFZ classifier matched the *BCOR* ITD tumor to “CNS high grade neuroepithelial tumor with *BCOR* alteration (CNS HGNET-*BCOR*)” with a high score (0.99). The *BCORL1*-fusion tumor was interpreted by the DKFZ classifier as a ‘no match,’ although it was also classified as CNS HGNET-*BCOR* with a low score (0.44) (Fig. 1, Additional file 3 Table S3). Thus it is likely that these tumors may belong to a new entity within the unclassified heterogeneous high grade neuroectodermal/glial tumors of children [30]. An HDAC inhibitor may potentially be effective for tumors with activated *BCOR/BCORL1*.

Much less is known about the *FOXO1-STK24* fusion found in another ST-EPN (EP57). *FOXO1* is a transcription factor that is involved in the maintenance of cellular homeostasis [36]. *PAX3-FOXO1* fusion, which acts as a highly activated transcription factor, is found in 60% of alveolar rhabdomyosarcomas [36]. *STK24* (also known as *MST3*) is a serine-threonine kinase that functions upstream of the mitogen-activated kinase (MAK) signaling pathway. *STK24/MST3* is overexpressed in breast cancers and promotes proliferation and tumorigenicity [30]. Recurrent mutations or fusions of *STK24* have not been reported. The DKFZ classifier found no match for this ST-EPN tumor (classified as PFB, score = 0.44). Interestingly, this tumor showed copy number oscillation compatible with chromothripsis on chromosomes 13, on which *FOXO1* and *STK24* are located, strongly suggesting that this may be the mechanism underlying the gene fusion. Both *FOXO1* and *STK24* were overexpressed in EP57 (Additional file 10 Figure S8), suggesting that either of them may carry an oncogenic property. Although a detailed study of individual cases is beyond the scope of this paper, this tumor may warrant further investigation.

None of the other *RELA* fusion-negative ST-EPN were classifiable even with the DKFZ classifier. In summation, our findings suggest that *RELA/YAP1* fusion-negative ST-EPNs may be a heterogeneous group of tumors that consist of a variety of mutations or rare fusion genes, which are unlikely to belong to a single category. Further studies using a vast number of tumors may help in clarifying whether tumors with similar genetic changes and/or DNA methylation profiles truly define a new tumor entity. Considering the high homogeneity of *RELA*-fusion positive ST-EPNs, it is doubtful whether these are biologically equivalent to ependymoma. According to the latest WHO Classification [8], ependymomas are primarily diagnosed via histology. As such, they may be diagnosed as ependymomas, at least for the time being. Nonetheless, it is important to be aware that histologically diagnosed RELA-fusion negative ependymomas may have a biology which is different from that of quintessential RELA-fusion positive ependymomas. Further molecular classification and incorporation into future WHO Classification criteria is warranted.

In contrast to a previous large series, no significant association between the presence of *C11orf95-RELA* fusion and patient survival was noticed in our series [25]. Furthermore, RELA fusion status was reportedly not related to a significant difference in the survival of ST-EPN patients [9]. In addition, the rate of GTR in RELA fusion-positive ST-EPN was not statistically significant compared to that in RELA fusion-negative ST-EPN (*p* = 0.55) in our cohort. The impact of *C11orf95-RELA* fusion on patient survival needs to be further investigated. These findings may reflect the fact that *RELA* fusion-negative ST-EPNs are a biologically heterogeneous group of tumors. Interestingly, median progression-free or overall survival was not reached for *C11orf95-RELA* fusion positive ST-EPNs. Other proposed prognostic molecular markers of ependymomas include *TERT* and *EZH2* expression [18, 21, 31]. Although we confirmed elevated *EZH2* and *TERT* expression in *RELA* fusion-positive ST-EPNs, they were not associated with patient survival. Nonetheless, it may be of interest that *TERT* mRNA expression was elevated in *RELA* fusion-positive ST-EPNs, to an extent which far exceeded that in glioblastomas with *TERT* promoter mutations (Additional file 9 Figure S4b). None of the ST- or PF-EPNs in this cohort carried the *TERT* promoter mutation (data not shown). This phenomenon has also been described elsewhere [10]. Costelo-Branco et al., found that the methylation status of some CpG sites upstream of transcription starting site of *TERT,* were positively correlated with *TERT* mRNA expression in childhood malignant brain tumors and were also associated with the prognosis of patients with PF ependymoma [5]. Although neither *TERT* mRNA expression nor *TERT* UTSS methylation was associated with patient prognosis in this series, *TERT* UTSSs were highly methylated in the RELA fusion-positive ST-EPNs with elevated *TERT* mRNA expression. The mechanism of *TERT* upregulation appears to be complex and warrants further investigation.

We validated the proposed molecular classification of PF-EPN for efficacy in predicting clinical characteristics including that of patient survival. The 450 K analysis accurately classified the published reference PF-EPN dataset, confirming the robustness of the analysis. PFA showed a minor but significant increase in methylation levels and distinct methylation profiles when compared to PFB (Fig. 2). With a few exceptions, PFA patients were mostly infants and the ages of the PFB patients were significantly higher than those of PFA (Additional file 14 Figure S3a). PFA tumors showed significantly more lateral extension compared to PFB, most of which were medially located (Additional file 14 Figure S3b). DKFZ classifier results were mostly consistent with our analysis with a few exceptions. Two PFAs showed no match. One PFB (EP96) was classified as pituitary adenoma and another PFB (EP86) as myxopapillary ependymoma. These classifications were not compatible with their histology or location.

Our multivariate analysis using Cox regression showed that the PFA subgroup was the only molecular marker which was independently associated with patient PFS and OS among all ependymomas. Among PF-EPN, PFA patients showed significantly shorter PFS and OS compared to PFB patients. These findings corroborated previous reports [19, 29] and consolidated the significance of proposed molecular classification, indicating that PFA and PFB may be biologically distinct subgroups of PF-EPN. The important clinical implication of the PFA/PFB classification is its potential to aid therapeutic decision making. Based on the results of a study conducted on a large series of PF-EPN, Ramaswamy suggested that a substantial proportion of totally resected PFB patients may be treated with surgery alone, without radiotherapy [29]. Although this suggestion needs to be tested in a randomized clinical trial, it is evident that molecular classification may play an important role in the clinical management of ependymomas. Although resection rate was not significantly associated with survival in our survival analysis, there was a tendency for gross total resection (GTR) to predict longer survival (Additional file 15 Figure S9). This may be due to the relatively small number of cases screened in the study. Retrospectively, the extent of resection although determined locally was not centrally reviewed which may be a limitation of the multi-institutional nature of the study. Data from the Collaborative Ependymoma Research Network (CERN), which was also a multi-institutional study, did not indicate statistically significant differences in PFA survival due to resection rate [29]. A prospective clinical trial for ST-and PF-EPN with molecular classification and a standardized central review for the extent of resection is on-going in Japan Children’s Cancer Group (JCCG).

In spite of its usefulness, a practical problem associated with methylation classification, is its cost as well as limited availability as a routine diagnostic test. To overcome this issue, a simplified methylation test was developed to determine PFA/PFB for PF-EPNs by examining the 3 most highly methylated regions in PFA via pyrosequencing and rigorously validating it by using an extended PF-EPN cohort of 123 PF-EPNs which combined our cases and an independent set of samples from Toronto (Results and Fig. 5). With this novel assay, we were able to diagnose PFB with 100% specificity. We also confirmed the efficacy of anti-H3K27me3 immunohistochemistry, recently reported by Panwalkar et al. [26], by predicting PFB with 100% specificity in a selected Japanese cohort of 44 PF-EPNs (Fig. 5 ). It may be noteworthy, that the proposed cutoff of 80% for H3K27me3 immunohistochemistry, though appropriate, may prove to be somewhat counter-intuitive for judging reduced PFA expression. Among the 4 PFBs examined via both methods, certain single cases were misclassified as PFA in each method. Although methylation assessment at individual CpG sites has its own limitation such as potential heterogeneity of methylation across CpGs as well as masking by co-existing non-neoplastic cells, these assays may serve as clinically applicable techniques for rapid molecular classification of PF-EPN, which are also suitable for risk-grouping in clinical trials. Hopefully, these may lead to better treatment decision making for the ependymoma patients in the future.

It has been suggested that the presence of 1q gain is associated with poor prognosis in ependymomas [13, 16, 24, 25]. A large cohort study indicated significant differences in PFS between patients with and without 1q gain in both PFA and PFB, whereas significantly shorter overall survival in patients with 1q gain was seen only in PFA patients but not in PFB or ST-EPN-*RELA* patients [6, 24, 25]. In our cohort, 1q gain was highly enriched in a subset of PFA, while only PFB had 1q gain (Fig. 2). PFA patients with 1q gain were older at onset (*p* < 0.001; Fig. 3a) and exhibited significantly shorter PFS than those without 1q gain (*p* = 0.016, Fig. 4e). However, there was no significant difference of OS between those patients (*p* = 0.51, Fig. 4f). The reason for discrepancies related to the impact of 1q gain on OS of PF-EPN between our study and others is currently unknown. It has been recently proposed that PFA and PFB may further be divided into 9 or 5 subgroups [6, 24]. Although our cohort was too small to validate such subgrouping, it is likely that PF-EPN are a heterogeneous group of tumors. The significance of molecular markers/subgroups in patient prognosis needs to be examined by a prospective study.

Our study demonstrated that histopathological diagnosis of ependymomas such as ST-EPN is often challenging. Eight locally diagnosed ST-EPNs were re-classified as non-EPN tumors following a pathology review. None of them carried *RELA*-fusion. The significance of WHO grading of ependymomas is highly controversial [7, 25]. On the other hand, our histopathological review classified most PFA as WHO grade III, and PFB as WHO grade II. Current WHO Classification bases the diagnosis of ependymomas solely on the histopathology of tumors. Thus the role of histopathology needs to be revisited.

## Conclusion

Our results showed that *C11orf95-RELA* fusion is a unique and highly specific diagnostic marker for ST-EPN. However, histologically verified *RELA* fusion- or *YAP1* fusion-negative ST-EPN also exists. These cases were neither histologically nor molecularly subependymomas, and thus did not fall into any of the 3 proposed molecular groups of ST-EPN [25]. They appear to be a very heterogeneous group of tumors distinct from the RELA fusion-positive ST-EPN, and are unlikely to fall into a single category. However, most if not each one of them may rather belong to different, possibly new entities, considering the DKFZ classifier results. A more thorough implementation of molecular diagnosis may hopefully resolve unanswered questions. While the definition of ependymoma awaits future discussion, it is clear that histology alone may not be sufficient for a perfect definition of this disease. Although the true clinical impact of molecular classification, especially for therapeutic decision making, needs to be determined in a prospective clinical trial, our study clearly demonstrated that molecular classification may hold the key to future management of ependymomas.

## Additional files

Additional file 1: **Table S1.** Primer sequences and pyrosequencing assays (XLSX 14 kb)

Additional file 2: **Table S2.** Candidate probes in screening set for pyrosequencing assay (XLSX 11 kb)

Additional file 3: **Table S3.** List of cases (XLSX 66 kb)

Additional file 4: **Figure S5.** Methylation percentages for *CRIP1*, *DRD4*, and *LBX2* in datasets. (a) Methylation percentages for the three genes in the original dataset of JPMNG and SickKids. (b) Methylation percentages for the three genes in the validation dataset. The dashed lines denote the thresholds determined by likelihood ratio in the training process. (TIF 6463 kb)

Additional file 5: **Table S4.** RNA sequencing results (XLSX 51 kb)

Additional file 6: **Figure S1.** (a) Electropherograms of novel fusion transcripts detected in ST-EPNs. (b) Copy number analysis of EP57 showing copy number oscillation in chromosome 6 and 13 (The DKFZ Classifier output, molecularneuropathology.org). (TIF 7893 kb)

Additional file 7: **Figure S6.** (a) (b) Copy number plots of EP33 showing loss of upstream exon2 of *RELA.* (c) Immunohistochemical staining of L1 cell adhesion molecule (L1CAM) presents strong positivity in EP33. (TIF 9820 kb)

Additional file 8: **Figure S7.** Histological features in *RELA*-negative/*YAP1*-negative supratentorial ependymoma cases. EP3 (*EP300*-*BCORL1* fusion-positive) exhibits typical findings of anaplastic ependymoma, including hypercellularity, perivascular pseudorosettes (a), calcification (arrows, b) and high MIB-1 labeling index (c). In EP57 (*FOXO1*-*STK24* fusion-positive), perivascular pseudorosettes (d), calcification (arrows, e), microcyst formation (e), vacuolated cells (f), GFAP-positive cells (g), EMA positive reaction (h) and low MIB-1 labeling index (i) were observed. (TIF 58932 kb)

Additional file 9: **Figure S4.** Box plots showing *EZH2* (a), *TERT* expression (b), and methylation status of upstream transcription starting site of *TERT* (c). Significant upregulation of these markers in *C11orf95-RELA* fusion positive EPNs was observed. (TIF 2376 kb)

Additional file 10: **Figure S8.** Expression data of (a) *EP300,* (b) *BCORL1,* (c) *FOXO1,* and (d) *STK24* among supratentorial ependymomas. EP3 and EP57 show by far the highest expression levels of *BCORL1* and *FOXO1* among all ST-EPNs, respectively. (TIF 5041 kb)

Additional file 11: **Figure S2.** Classification of posterior fossa (PF-EPN) and spinal ependymomas (SP-EPN) using genome-wide methylation profiling. A heatmap analyzed by 3932 probes that showed high standard deviations (SD > 0.25) on CpG islands for unsupervised hierarchical clustering of 72 centrally-diagnosed posterior and spinal ependymoma samples shows that nearly all spinal tumors except one (EP114) were clustered with posterior fossa PFB. The following information is indicated below the heatmap: tumor location, a pattern of PF tumors extension, pathological grading, the presence of 1q gain, age at onset, and the DKFZ classifier result. (TIF 6031 kb)

Additional file 12: **Table S6.** Univariate and multivariate analysis of ST and PF ependymomas in PFS and OS. (DOCX 44 kb)

Additional file 13: **Table S5.** Validation results of PF-EPN subgroup prediction rule candidates. (DOCX 16 kb)

Additional file 14: **Figure S3.** Comparison of clinical characteristics between posterior fossa PFA and PFB. (a) Box plot showing the distribution of the patients’ age at onset. (b-d) Mosaic plot for tumor location, pathological grading, and resection rate in posterior fossa tumors. Comparison of clinical characteristics of PFA stratified by the presence of 1q gain. (e) Box plot showing the distribution of the patients’ age at onset. (f-h) Mosaic plot of tumor location, dissemination at onset, and resection rate in PFA tumors. (TIF 6273 kb)

Additional file 15: **Figure S9** Progression-free survival (PFS, a, c, e, g, i) and overall survival (OS, b, d, f, h, j) of histologically verified all-EPNs (a, b), ST-EPNs (c, d), PF-EPNs (e, f), PFA-EPNs (g, h), and PFB-EPNs (i, j) stratified according to the extent of resection. (TIF 32224 kb)

Additional file 16: The Japan Pediatric Molecular Neuro-Oncology Group (JPMNG): participating centers and departments. (DOCX 17 kb)

## Abbreviations

EPN: Ependymoma
FISH: Fluorescent in situ hybridization
GBM: Glioblastoma
PF-EPN: Gosterior fossa ependymoma
RT-PCR: Reverse transcription- polemerase chain reaction
SP-EPN: Spinal ependymoma
ST-EPN: Supratentorial ependymoma
