# Supplementary figures and images for: Significance of molecular classification of ependymomas: C11orf95-RELA fusion-negative supratentorial ependymomas are a heterogeneous group of tumors
Source: Acta Neuropathol Commun. 2018 Dec 4;6:134. doi: 10.1186/s40478-018-0630-1 (PMC6278135; doi:10.1186/s40478-018-0630-1)

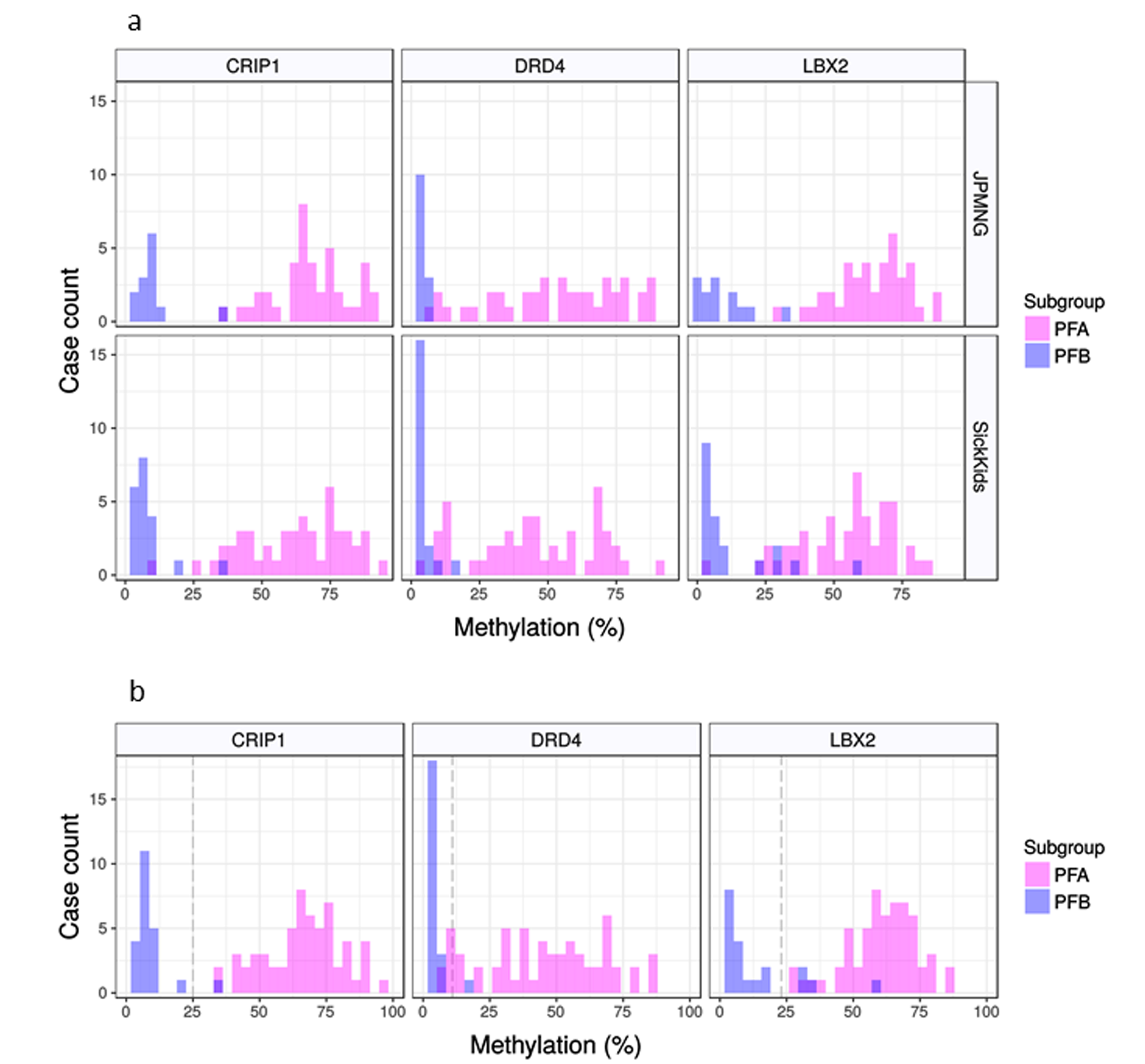

Supplement: Supplementary file 4 — Figure S5. Methylation percentages for CRIP1, DRD4, and LBX2 in datasets. (a) Methylation percentages for the three genes in the original dataset of JPMNG and SickKids. (b) Methylation percentages for the three genes in the validation dataset. The dashed lines denote the thresholds determined by likelihood ratio in the training process. (TIF 6463 kb) [file 40478_2018_630_MOESM4_ESM.tif]

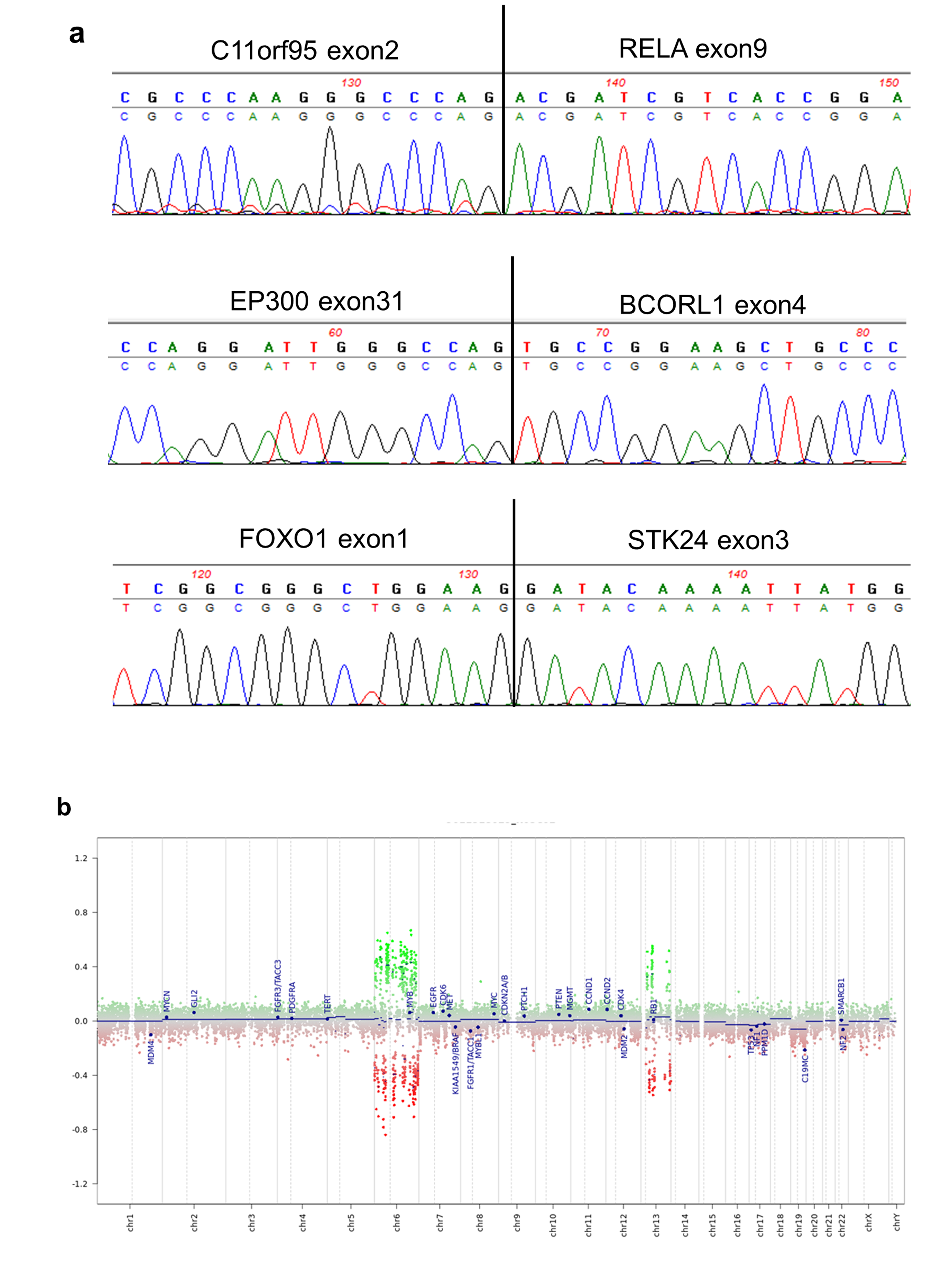

Supplement: Supplementary file 6 — Figure S1. (a) Electropherograms of novel fusion transcripts detected in ST-EPNs. (b) Copy number analysis of EP57 showing copy number oscillation in chromosome 6 and 13 (The DKFZ Classifier output, molecularneuropathology.org). (TIF 7893 kb) [file 40478_2018_630_MOESM6_ESM.tif]

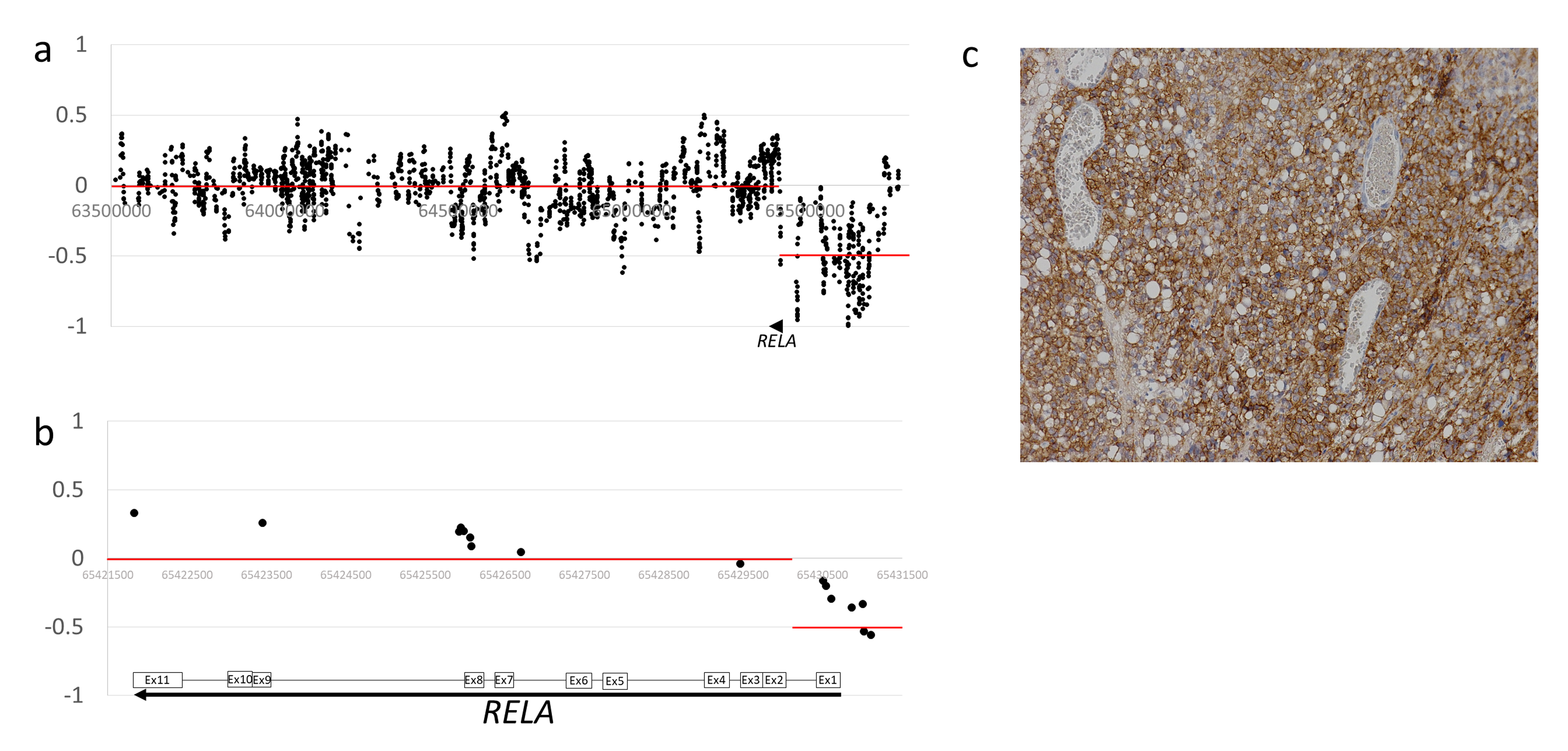

Supplement: Supplementary file 7 — Figure S6. (a) (b) Copy number plots of EP33 showing loss of upstream exon2 of RELA. (c) Immunohistochemical staining of L1 cell adhesion molecule (L1CAM) presents strong positivity in EP33. (TIF 9820 kb) [file 40478_2018_630_MOESM7_ESM.tif]

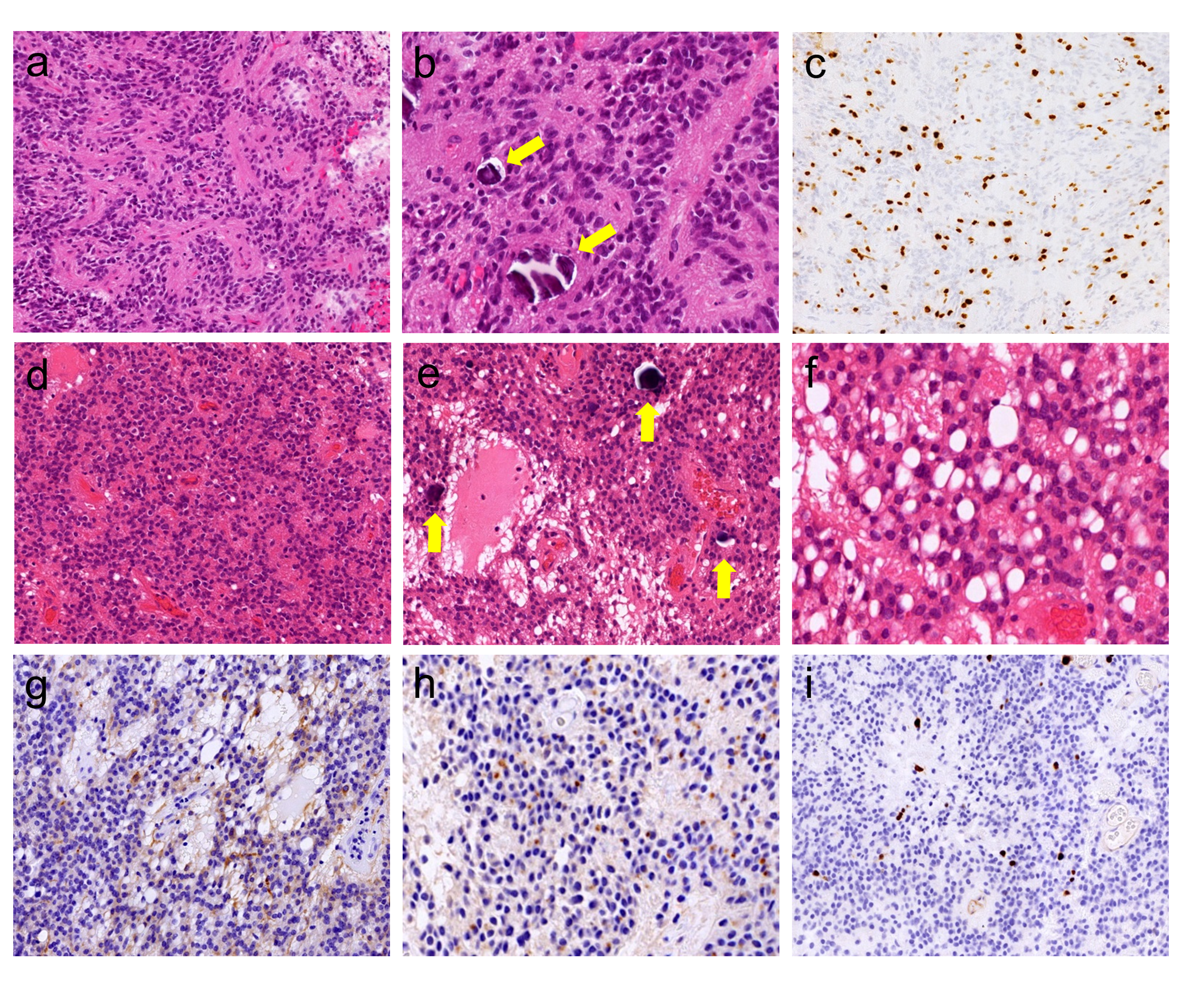

Supplement: Supplementary file 8 — Figure S7. Histological features in RELA-negative/YAP1-negative supratentorial ependymoma cases. EP3 (EP300-BCORL1 fusion-positive) exhibits typical findings of anaplastic ependymoma, including hypercellularity, perivascular pseudorosettes (a), calcification (arrows, b) and high MIB-1 labeling index (c). In EP57 (FOXO1-STK24 fusion-positive), perivascular pseudorosettes (d), calcification (arrows, e), microcyst formation (e), vacuolated cells (f), GFAP-positive cells (g), EMA positive reaction (h) and low MIB-1 labeling index (i) were observed. (TIF 58932 kb) [file 40478_2018_630_MOESM8_ESM.tif]

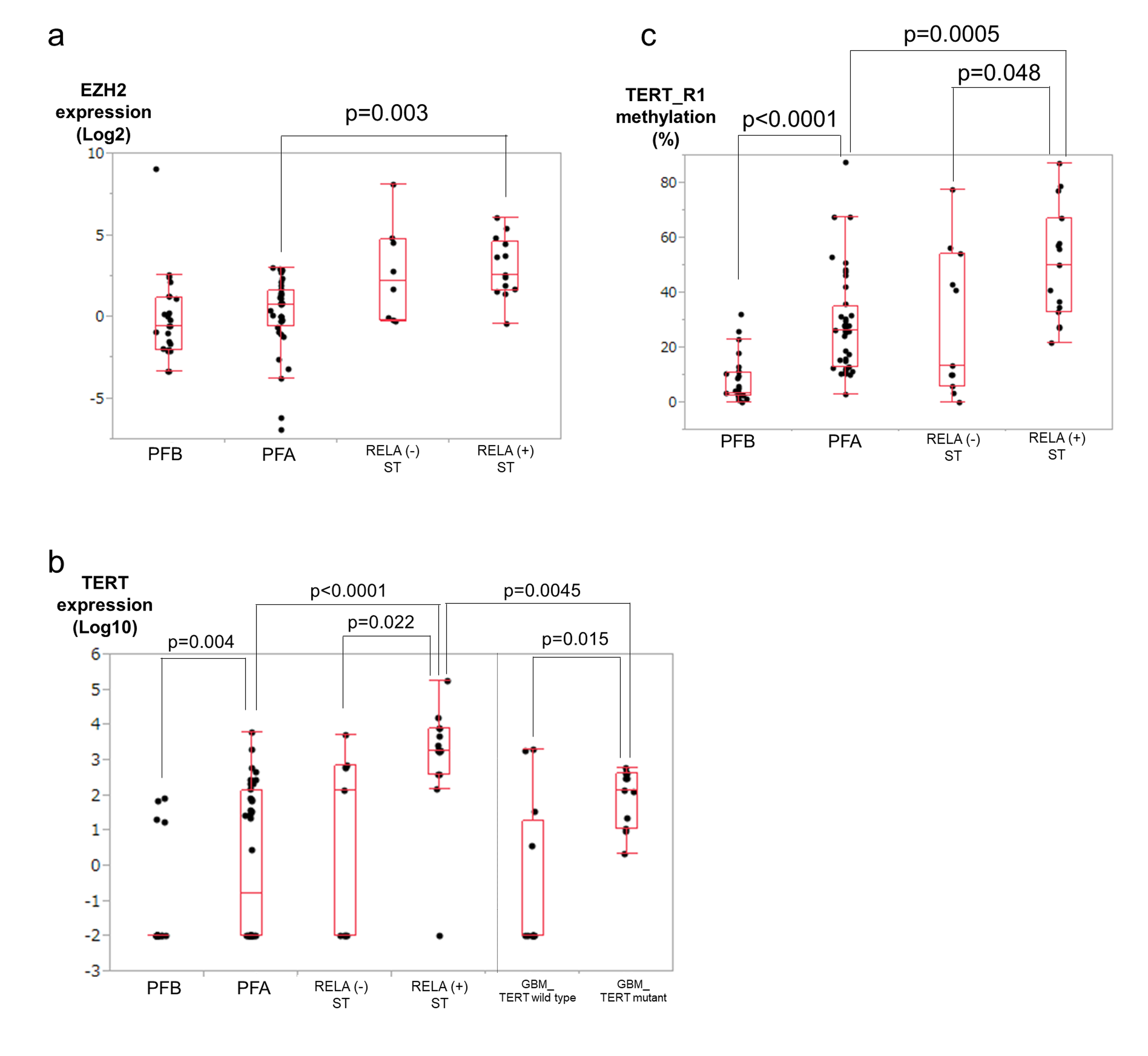

Supplement: Supplementary file 9 — Figure S4. Box plots showing EZH2 (a), TERT expression (b), and methylation status of upstream transcription starting site of TERT (c). Significant upregulation of these markers in C11orf95-RELA fusion positive EPNs was observed. (TIF 2376 kb) [file 40478_2018_630_MOESM9_ESM.tif]

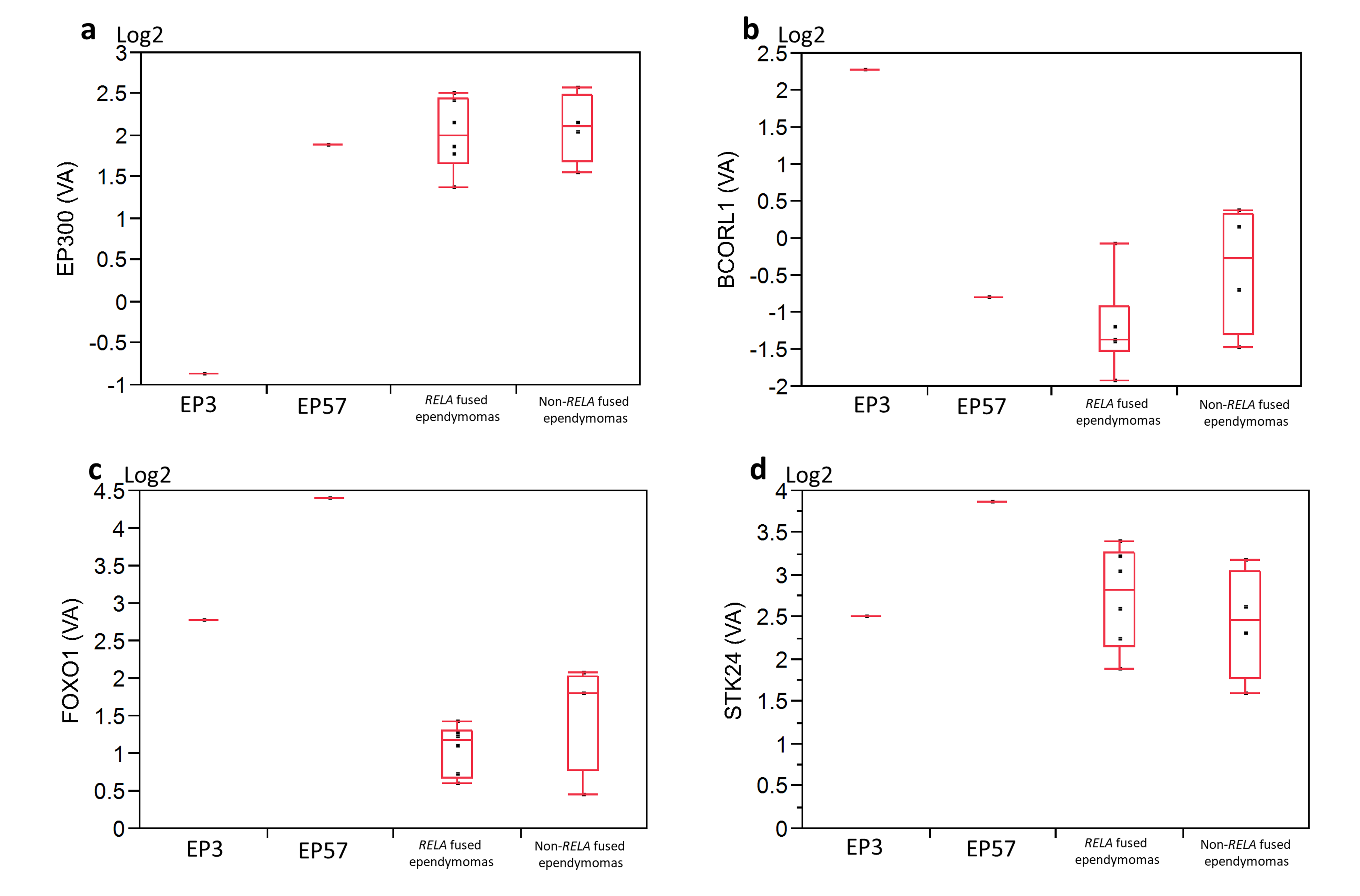

Supplement: Supplementary file 10 — Figure S8. Expression data of (a) EP300, (b) BCORL1, (c) FOXO1, and (d) STK24 among supratentorial ependymomas. EP3 and EP57 show by far the highest expression levels of BCORL1 and FOXO1 among all ST-EPNs, respectively. (TIF 5041 kb) [file 40478_2018_630_MOESM10_ESM.tif]

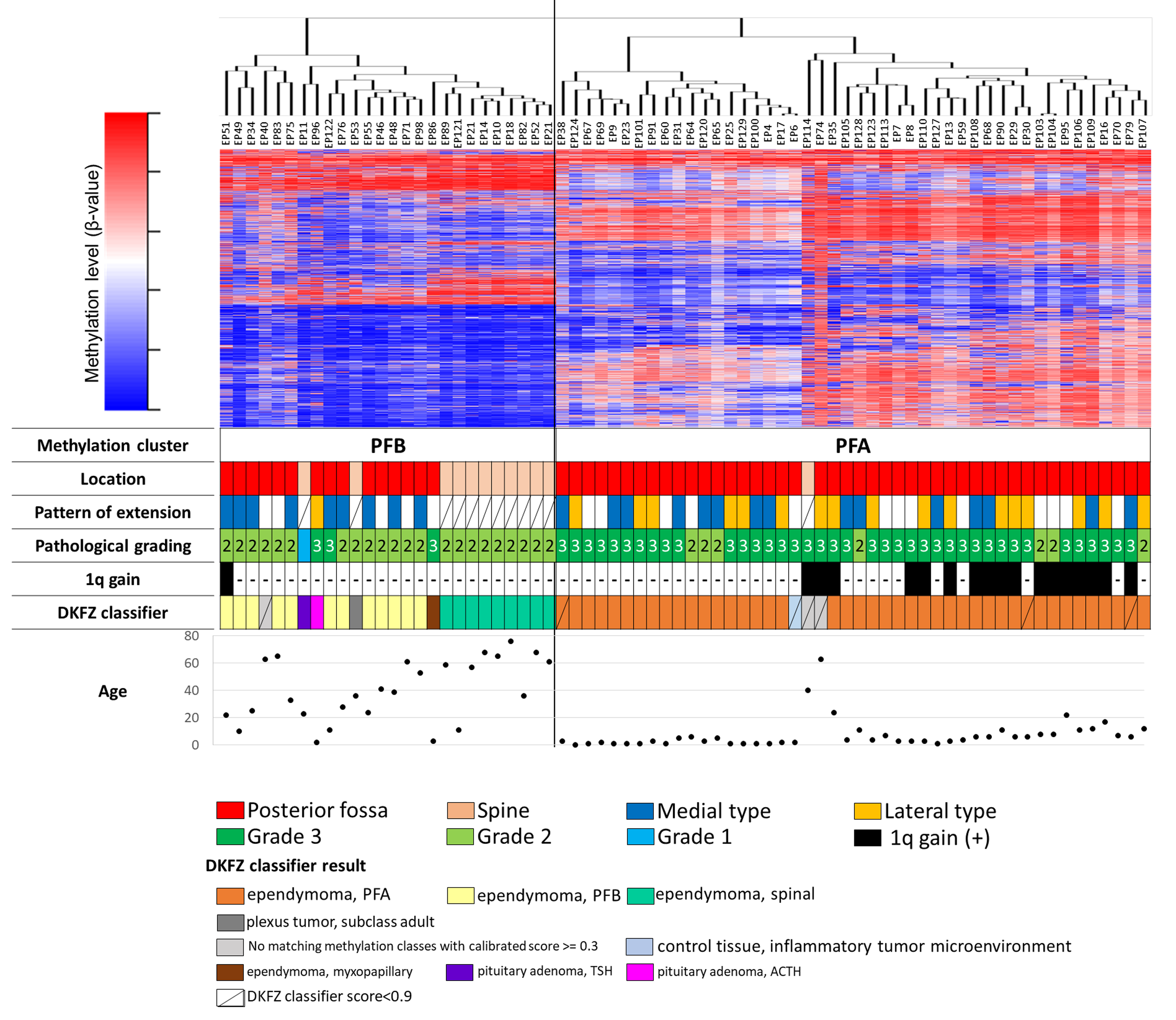

Supplement: Supplementary file 11 — Figure S2. Classification of posterior fossa (PF-EPN) and spinal ependymomas (SP-EPN) using genome-wide methylation profiling. A heatmap analyzed by 3932 probes that showed high standard deviations (SD > 0.25) on CpG islands for unsupervised hierarchical clustering of 72 centrally-diagnosed posterior and spinal ependymoma samples shows that nearly all spinal tumors except one (EP114) were clustered with posterior fossa PFB. The following information is indicated below the heatmap: tumor location, a pattern of PF tumors extension, pathological grading, the presence of 1q gain, age at onset, and the DKFZ classifier result. (TIF 6031 kb) [file 40478_2018_630_MOESM11_ESM.tif]

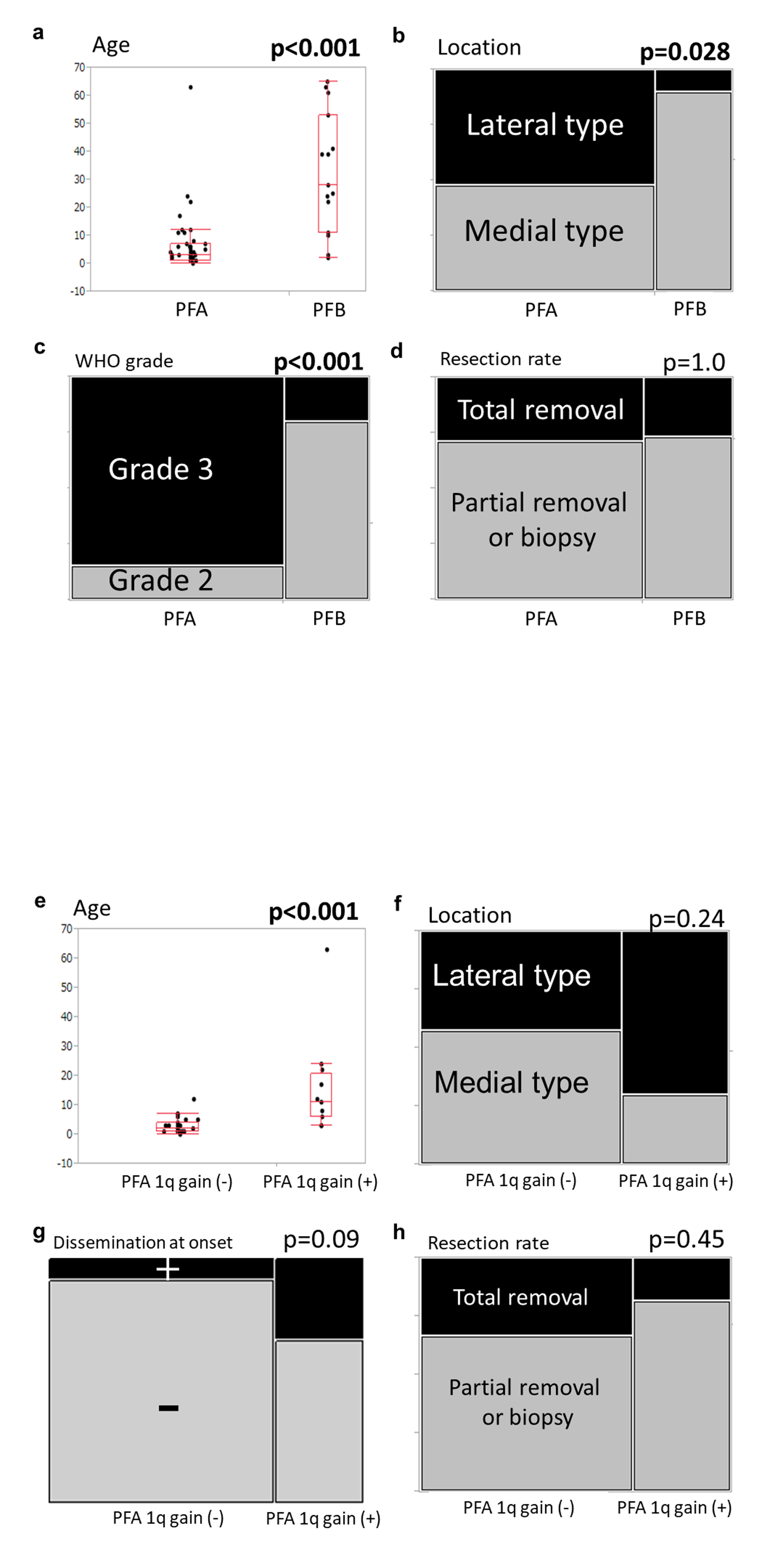

Supplement: Supplementary file 14 — Figure S3. Comparison of clinical characteristics between posterior fossa PFA and PFB. (a) Box plot showing the distribution of the patients’ age at onset. (b-d) Mosaic plot for tumor location, pathological grading, and resection rate in posterior fossa tumors. Comparison of clinical characteristics of PFA stratified by the presence of 1q gain. (e) Box plot showing the distribution of the patients’ age at onset. (f-h) Mosaic plot of tumor location, dissemination at onset, and resection rate in PFA tumors. (TIF 6273 kb) [file 40478_2018_630_MOESM14_ESM.tif]
