# Supplementary material for: Significance of molecular classification of ependymomas: C11orf95-RELA fusion-negative supratentorial ependymomas are a heterogeneous group of tumors
Source: Acta Neuropathol Commun. 2018 Dec 4;6:134. doi: 10.1186/s40478-018-0630-1 (PMC6278135; doi:10.1186/s40478-018-0630-1)
Supplement: Supplementary file 12 — Table S6. Univariate and multivariate analysis of ST and PF ependymomas in PFS and OS. (DOCX 44 kb) [file 40478_2018_630_MOESM12_ESM.docx]

| **Supplementary table 6.** Univariate analysis of progression free survival (PFS) and Overall survival (OS) among supratentorial (ST) tumors  **Univariate analysis of PFS among ST tumors** | | | |
| --- | --- | --- | --- |
| **Variable** | **Hazard ratio (HR)** | **95% confidence  interval for HR** | **p-value** |
| Incomplete resection | 0.95 | 0.16-17.9 | 0.96 |
| WHO grade3 | 1618338.2 | 0.68-0.69 | 0.09 |
| C11orf95-RELA fusion | 0.47 | 0.11-2.35 | 0.47 |
| EZH2 high expression | 2.69 | 0.39-52.87 | 0.33 |
| TERT high expression | 0.95 | 0.20-4.86 | 0.94 |
| TERT UTSS high methylation | 1468381.1 | 0.33-0.34 | 0.23 |
| Local radiation therapy>=50Gy | 0.49 | 0.11-2.11 | 0.32 |
| Chemotherapy | 1.01 | 0.20-4.16 | 0.98 |
|  |  |  |  |
|  |  |  |  |
| **Univariate analysis of OS among ST tumors** | | | |
| **Variable** | **Hazard ratio (HR)** | **95% confidence  interval for HR** | **p-value** |
| Incomplete resection | 0.47 | 0.06-9.70 | 0.47 |
| WHO grade3 | 1561452.2 | 0.27-0.28 | 0.26 |
| C11orf95-RELA fusion | 1.06 | 0.13-21.68 | 0.95 |
| EZH2 high expression | 9838994.3 | 0.15-0.16 | 0.25 |
| TERT high expression | 88889779.4 | 0.36-0.37 | 0.16 |
| TERT UTSS high methylation | 1576137.6 | 0.16-0.17 | 0.35 |
| Local radiation therapy>=50Gy | 1.42 | 0.13-31.13 | 0.77 |
| Chemotherapy | 4.69 | 0.41-107.1 | 0.20 |
|  |  |  |  |

| **Supplementary table 6.** Univariate analysis of progression free survival (PFS) and Overall survival (OS) among posterior fossa (PF) tumors  **Univariate analysis for PFS among PF tumors** | | | |
| --- | --- | --- | --- |
| **Variable** | **Hazard ratio (HR)** | **95% confidence  interval for HR** | **p-value** |
| Incomplete resection | 1.21 | 0.55-2.92 | 0.64 |
| **WHO grade3** | **1.98** | **0.87-5.04** | **0.010** |
| **PFA** | **2.41** | **0.99-7.21** | **0.053** |
| **1q gain** | **3.81** | **1.54-8.64** | **0.005** |
| EZH2 high expression | 1.27 | 0.57-2.83 | 0.55 |
| TERT high expression | 1.36 | 0.60-3.00 | 0.45 |
| TERT UTSS high methylation | 0.86 | 0.41-1.87 | 0.71 |
| Local radiation therapy>=50Gy | 0.55 | 0.25-1.18 | 0.55 |
| Chemotherapy | 1.15 | 0.52-2.46 | 0.73 |
|  |  |  |  |
| **Multivariate analysis for PFS among PF tumors** | |  |  |
| **Variable** | **Hazard ratio (HR)** | **95% confidence  interval for HR** | **p-value** |
| WHO grade3 | 0.10 | 0.87-5.05 | 0.10 |
| **PFA** | **2.41** | **0.99-7.21** | **0.053** |
| **1q gain** | **3.81** | **1.54-8.64** | **0.005** |
|  |  |  |  |
| **Univariate analysis for OS among PF tumors** | | |  |
| **Variable** | **Hazard ratio (HR)** | **95% confidence  interval for HR** | **p-value** |
| Incomplete resection | 1.59 | 0.56-5.66 | 0.40 |
| **WHO grade3** | **5.24** | **1.48-33.2** | **0.007** |
| **PFA** | **9.04** | **1.85-163.0** | **0.003** |
| 1q gain | 1.86 | 0.52-5.31 | 0.31 |
| EZH2 high expression | 1.61 | 0.60-4.51 | 0.34 |
| TERT high expression | 0.81 | 0.28-2.19 | 0.68 |
| TERT UTSS high methylation | 1.41 | 0.55-4.10 | 0.47 |
| Local radiation therapy>=50Gy | 1.14 | 0.41-3.42 | 0.80 |
| Chemotherapy | 1.13 | 0.40-3.04 | 0.80 |
|  |  |  |  |
| **Multivariate analysis for OS among PF tumors** | |  |  |
| **Variable** | **Hazard ratio (HR)** | **95% confidence  interval for HR** | **p-value** |
| WHO grade3 | 1.92 | 0.44-16.8 | 0.43 |
| PFA | 5.43 | 0.62-120.6 | 0.14 |
