# Supplementary material for: Significance of molecular classification of ependymomas: C11orf95-RELA fusion-negative supratentorial ependymomas are a heterogeneous group of tumors
Source: Acta Neuropathol Commun. 2018 Dec 4;6:134. doi: 10.1186/s40478-018-0630-1 (PMC6278135; doi:10.1186/s40478-018-0630-1)
Supplement: Supplementary file 13 — Table S5. Validation results of PF-EPN subgroup prediction rule candidates. (DOCX 16 kb) [file 40478_2018_630_MOESM13_ESM.docx]

### Supplementary Table S5: Validation results of the PF-EPN subgroup prediction rule candidates

| Prediction rule | Dataset | Accuracy | Sensitivity for PFB | Specificity for PFB |
| --- | --- | --- | --- | --- |
| IF all of genes suggest PFB, THEN classify a case as PFB | Training | 0.926829 | 0.727273 | 1.000000 |
| IF all of genes suggest PFB, THEN classify a case as PFB | Validation | 0.939024 | 0.772727 | 1.000000 |
| IF a majority of genes suggest PFB, THEN classify a case as PFB | Training | 0.975610 | 1.000000 | 0.966667 |
| IF a majority of genes suggest PFB, THEN classify a case as PFB | Validation | 0.987805 | 0.954545 | 1.000000 |
| IF any of genes suggest PFB, THEN classify a case as PFB | Training | 0.902439 | 1.000000 | 0.866667 |
| IF any of genes suggest PFB, THEN classify a case as PFB | Validation | 0.939024 | 1.000000 | 0.916667 |
