# Supplementary material for: Significance of molecular classification of ependymomas: C11orf95-RELA fusion-negative supratentorial ependymomas are a heterogeneous group of tumors
Source: Acta Neuropathol Commun. 2018 Dec 4;6:134. doi: 10.1186/s40478-018-0630-1 (PMC6278135; doi:10.1186/s40478-018-0630-1)
Supplement: Supplementary file 16 — The Japan Pediatric Molecular Neuro-Oncology Group (JPMNG): participating centers and departments. (DOCX 17 kb) [file 40478_2018_630_MOESM16_ESM.docx]

**Significance of molecular classification of ependymomas: C11orf95-RELA fusion-negative supratentorial ependymomas are a heterogeneous group of tumors**

Kohei Fukuoka, Yonehiro Kanemura, Tomoko Shofuda, et al.

# **Supplementary material: The Japan Pediatric Molecular Neuro-Oncology Group (JPMNG): participating centers and departments**

Department of Neurosurgery, Hokkaido Medical Center for Child Health and Rehabilitation, Sapporo, Japan

Department of Neurosurgery, Hokkaido University Graduate School of Medicine, Sapporo, Japan

Department of Neurosurgery, Tohoku University Graduate School of Medicine, Sendai, Japan

Department of Neurosurgery, Gunma University Graduate School of Medicine, Maebashi, Gunma, Japan

Department of Pathology, Gunma University Hospital, Maebashi, Gunma, Japan

Department of Neurosurgery, Teikyo University Hospital, Mizonokuchi, Kanagawa, Japan

Department of Neuro-Oncology/Neurosurgery, Saitama Medical University International Medical Center, Hidaka, Saitama, japan

Department of Pathology, Saitama Medical University, Saitama, Japan

Department of Neurosurgery and Neuro-oncology, National Cancer Center Hospital, Tokyo, Japan

Division of Brain Tumor Translational Research, National Cancer Center Research Institute, Tokyo, Japan

Department of Neurosurgery, Juntendo University, Tokyo, Japan

Department of Pediatrics, The Jikei University School of Medicine, Tokyo, Japan

Department of Neurosurgery, Keio University School of Medicine, Tokyo, Japan

Department of Neurosurgery, Showa University School of Medicine, Tokyo, Japan

Department of Neurosurgery, Kyorin University Faculty of Medicine, Tokyo, Japan

Department of Laboratory Medicine and Pathology (Neuropathology), Tokyo Metropolitan Neurological Hospital, Tokyo, Japan.

Division of Hemato-Oncology and Regenerative Medicine, Kanagawa Children’s Medical Center, Yokohama, Kanagawa, Japan

Department of Neurosurgery, Shizuoka Children’s Hospital, Shizuoka, Japan

Department of Neurosurgery, Nagano Children's Hospital, Nagano, Japan

Department of Neurosurgery, Toyama University, Toyama, Japan

Department of Neurosurgery, Kanazawa Medical University, Kanazawa, Japan

Department of Neurosurgery, Kanazawa University School of Medicine, Kanazawa, Japan

Department of Neurosurgery, Fujita Health University Hospital, Toyoake, Aichi, Japan

Department of Pathology and Applied Neurobiology, Graduate School of Medical Science, Kyoto Prefectural University of Medicine, Kyoto, Japan

Department of Neurological Surgery, Wakayama Medical University, Wakayama, Japan

Department of Neurosurgery, Kansai Medical University, Hirakata, Osaka, Japan

Department of Neurosurgery, Osaka City University Graduate School of Medicine, Osaka, Japan

Department of Pediatric Hematology and Oncology and Department of Pediatric Neurosurgery, Osaka City General Hospital, Osaka, Japan

Department of Neurosurgery, Osaka University Graduate School of Medicine, Osaka, Japan

Department of Biomedical Research and Innovation, Institute for Clinical Research, Department of Neurosurgery, and Department of Pathology, Osaka National Hospital, National Hospital Organization, Osaka, Japan

Department of Pediatric Neurosurgery, Takatsuki General Hospital, Takatsuki, Osaka, Japan

Department of Neurosurgery, Faculty of Medicine, Kinki University, Osaka, Japan

Department of Neurosurgery, Tazuke Kofukai Foundation, Medical Research Institute and Kitano Hospital, Osaka, Japan

Department of Neurosurgery, Department of Pathology, and Department of Diagnostic Pathology, Hyogo Prefectural Kobe Children's Hospital, Kobe, Hyogo, Japan

Department of Diagnostic Pathology, Hyogo Cancer Center, Kobe, Hyogo, Japan

Department of Neurological Surgery, Okayama University Graduate School of Medicine, Dentistry and Pharmaceutical Sciences, Okayama, Japan

Department of Neurosurgery, University of Occupational and Environmental Health, Kitakyushu, Fukuoka, Japan

Department of Neurosurgery, Faculty of Life Sciences, Kumamoto University Graduate School, Kumamoto, Japan
